# 3D photopolymerized microstructured scaffolds influence nuclear deformation, nucleo/cytoskeletal protein organization, and gene regulation in mesenchymal stem cells

**DOI:** 10.1063/5.0153215

**Published:** 2023-09-07

**Authors:** Francesca Donnaloja, Manuela Teresa Raimondi, Letizia Messa, Bianca Barzaghini, Federica Carnevali, Emanuele Colombo, Davide Mazza, Chiara Martinelli, Lucia Boeri, Federica Rey, Cristina Cereda, Roberto Osellame, Giulio Cerullo, Stephana Carelli, Monica Soncini, Emanuela Jacchetti

**Affiliations:** 1 Department of Chemistry, Materials and Chemical Engineering “Giulio Natta,” Politecnico di Milano, Milan, Italy; 2 Department of Electronics, Information and Bioengineering, Politecnico di Milano, Milan, Italy; 3 Center of Functional Genomic and Rare Diseases, “V. Buzzi” Children's Hospital, 20154 Milan, Italy; 4 Istituto Scientifico Ospedale San Raffaele, Centro di Imaging Sperimentale, Milan, Italy; 5 Pediatric Research Center “Romeo ed Enrica Invernizzi,” Department of Biomedical and Clinical Sciences, University of Milan, Milan, Italy; 6 Institute for Photonics and Nanotechnologies—CNR, and Physics Department, Politecnico di Milano, Milan, Italy

## Abstract

Mechanical stimuli from the extracellular environment affect cell morphology and functionality. Recently, we reported that mesenchymal stem cells (MSCs) grown in a custom-made 3D microscaffold, the Nichoid, are able to express higher levels of stemness markers. In fact, the Nichoid is an interesting device for autologous MSC expansion in clinical translation and would appear to regulate gene activity by altering intracellular force transmission. To corroborate this hypothesis, we investigated mechanotransduction-related nuclear mechanisms, and we also treated spread cells with a drug that destroys the actin cytoskeleton. We observed a roundish nuclear shape in MSCs cultured in the Nichoid and correlated the nuclear curvature with the import of transcription factors. We observed a more homogeneous euchromatin distribution in cells cultured in the Nichoid with respect to the Flat sample, corresponding to a standard glass coverslip. These results suggest a different gene regulation, which we confirmed by an RNA-seq analysis that revealed the dysregulation of 1843 genes. We also observed a low structured lamina mesh, which, according to the implemented molecular dynamic simulations, indicates reduced damping activity, thus supporting the hypothesis of low intracellular force transmission. Also, our investigations regarding lamin expression and spatial organization support the hypothesis that the gene dysregulation induced by the Nichoid is mainly related to a reduction in force transmission. In conclusion, our findings revealing the Nichoid's effects on MSC behavior is a step forward in the control of stem cells via mechanical manipulation, thus paving the way to new strategies for MSC translation to clinical applications.

## I. INTRODUCTION

Mesenchymal stem cells (MSCs) are adult, multipotent cells, characterized by strong self-renewal, are widely available from different mesenchymal tissues, and can differentiate into multiple cell lineages (Mezey, 2022; Murphy *et al.*, 2013; Moon *et al.* 2018; and Le and Yao, 2017). MSCs can be isolated from the bone marrow of adult patients thereby avoiding ethical issues related to the therapeutic use of embryonic stem cells (Lo and Parham, 2009). All these characteristics, in addition to their low immunogenicity, make MSCs a very promising cell source for regenerative medicine applications (Cras *et al.* 2015; He *et al.* 2012; and Abd Elhalem *et al.*, 2018).

Understanding and controlling the potential of MSCs through cell signaling and biomedical engineering strategies, such as scaffolds, is still an open issue in terms of using MSCs in a clinical scenario (Pittenger *et al.*, 2019). One of the main challenges in stem cell clinical applications is how to control their fate without using chemical additives that can pose numerous risks for patients. Mechanical manipulation that exploits the high sensitivity of MSCs to environmental stimuli has been used to control cell fate and cell functionality (Tan *et al.*, 2020; Kim *et al.*, 2019; Luo and Seedhom, 2007; and Simmons *et al.* 2003).

The basic idea is to try to govern cell phenotypes simply by using a specific stiffness substrate or spatial confinement (Engler *et al.*, 2006; Olivares-Navarrete *et al.*, 2017; Tanaka *et al.*, 2017; Gerardo *et al.*, 2019; Ballester-Beltrán *et al.*, 2017; and Doolin *et al.*, 2020). A stiff substrate promotes osteogenic differentiation, an intermediate stiffness induces myogenic differentiation, and soft matrixes are neurogenic or even favor stem-like cellular phenotypes (Swift *et al.*, 2013). The extracellular environment affects the nuclear membrane permeability and the spatial organization of lamina and chromatin (Donnaloja *et al.*, 2019; Jean *et al.*, 2004; and Jahed *et al.*, 2014).

Nuclear envelope permeability is mechanically governed by the force-activation of nuclear pores, through which molecules are transported across the nucleus. An alteration in nuclear permeability directly impacts the transcription factor influx in the nucleus and gene expression (Gurdon, 2016; Elosegui-Artola *et al.*, 2017; García-González *et al.*, 2018; and Jacchetti *et al.*, 2021). Lamina is a network of intermediate filaments consisting in lamin proteins (named lamins A, B, and C type) that envelop the inner part of the nuclear membrane providing stability and protection for the whole nucleus. Lamin A/C structural arrangements change according to stimuli from the extra-cellular matrix and affect chromatin distribution, several nuclear functions such as DNA replication and repair, and stem cell potency and functions (Stiekema *et al.*, 2020; Donnaloja *et al.*, 2020; Shevelyov and Ulianov, 2019; Malashicheva and Perepelina, 2021; Lammerding *et al.*, 2004; Buxboim *et al.*, 2014; Dechat *et al.*, 2010; Seelbinder *et al.*, 2021; Shen and Niethammer 2020; Fritz *et al.*, 2019; Simon and Wilson, 2013; and Kind and van Steensel, 2010).

We developed the Nichoid 3D microscaffold (MOAB Srl, Milan, Italy) as a tool for stem cell culturing (Nava *et al.*, 2016; Raimondi *et al.*, 2014). The Nichoid consists of a micro-fabricated lattice substrate, produced by the two-photon polymerization technique, in an inert, biocompatible, and mechanically stable photoresin. It is characterized by a highly controlled spatial resolution (the thickness of each scaffold beam is 1.5 *μ*m) and by 90% porosity. The pore size, ranging from 10 to 30 *μ*m in the *xy-*plane, and 15 *μ*m along the *z* direction, enables cells to freely adhere in the three dimensions. It is suitable for cell culture and compared to the standard glass coverslip (the Flat), the Nichoid reduces cell adhesion in terms of the number and maturation of focal adhesions and maintains the MSC physiological functionality during cell expansion (Nava *et al.*, 2012; Nava *et al.*, 2016; Remuzzi *et al.*, 2020; and Jacchetti *et al.* 2021). Moreover, the Nichoid is optically accessible and enables real-time sample monitoring, so that the phenomena governing cellular functionality can be investigated in fixed and live cells (Parodi *et al.*, 2020; Jacchetti *et al.*, 2021).

In our previous papers, we computationally predicted how the Nichoid modulates the global influx of proteins in the nucleus in function of nuclear morphology (Jacchetti *et al.*, 2021), and we demonstrated the significant effects of the Nichoid on cellular geometry, nuclear localization of YAP/TAZ, cell proliferation, maintenance of cell stemness, and also on cell immunomodulatory function (Nava *et al.*, 2016; Carelli *et al.*, 2021; Rey *et al.*, 2020; Remuzzi *et al.*, 2020; Messa *et al.*, 2021; Testa *et al.*, 2023; and Barzaghini *et al.*, 2023).

Our previous results highlighted that because the Nichoid influences cellular functionality only by modulating its spatial organization, it is thus suitable for stem cell expansion culture in clinical applications and avoids exogenous factors that could be risky for the patient.

This paper furthers our previous results by investigating the Nichoid-related effects on the cellular elements mainly involved in the mechanotransduction pathway and gene regulation. We highlight how the reduction in intracellular forces induced by the Nichoid is sufficient to modulate the response of mechanotransducive cellular elements and to deregulate nearly 2000 genes.

## II. RESULTS

### A. The Nichoid affects actin organization, inducing a roundish nuclear shape

**FIG. 1.**
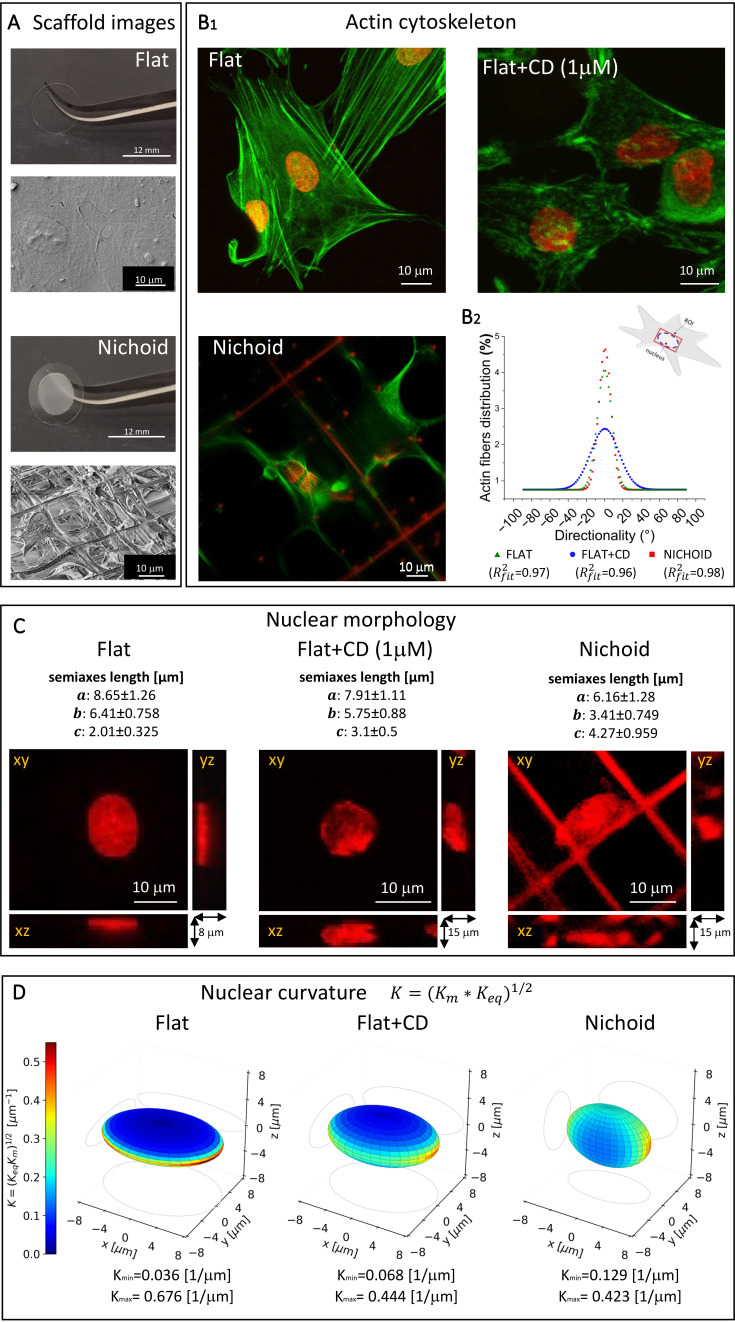
Morphology of MSCs grown in different culture conditions. (a) Photos of the cell culture systems: a glass coverslip substrate (Flat) and the 3D Nichoid microscaffold produced on the top of a glass coverslip, plus SEM images of cultured MSCs in the two conditions. (b) Analysis of the actin cytoskeletal organization. Representative fluorescence confocal images of MSCs grown on Flat substrate w/o 1 *μ*M cytochalasin-D, and in the Nichoid (b_1_). In green, the actin cytoskeleton (stained with phalloidin-FITC); in red, the cell nucleus (stained with Hoechst 33342). Analysis of the distribution of actin meshwork directly interacting with the cell nucleus (b_2_), investigated in a rectangular ROI circumscribing the nuclear ellipsis (as shown in the drawing). The images shown are maximum projections of z-stacks covering the entire cell volume. The number of analyzed cells was 214, 140, and 73 for Flat, Flat+CD, and Nichoid samples, respectively. Data were fitted with a Gaussian function. The goodness of fit is returned by the R square, which is higher than 0.96 for all the three cases investigated. (c) Lengths of nuclear semiaxes and representation of cell nuclei by orthogonal projection of z-stack images in Flat, Flat+CD, and Nichoid samples (from left to right panel, respectively). Nuclei were stained using the fluorescent dye Hoechst 33342. Note how as the intracellular tension decreases, the nucleus becomes rounder. Three independent samples of MSCs cultured on each scaffold were analyzed. The total number of analyzed cells was 235, 193, and 120 for Flat, Flat+CD, and Nichoid samples, respectively. Semiaxis data are reported as mean and standard deviation. (d) The nuclear shape model of MSCs grown on the Flat substrates (with and w/o cytochalasin-D treatment) and inside the Nichoid (from left to right, respectively). The color map represents the nuclear surface curvature defined as K = (K_eq_ × K_m_)^1/2^ (Bektas, 2017). The orthogonal projections of the ellipses are reported in the background. The maximum and the minimum curvature values are reported for each cell group.

To evaluate the Nichoid's impact on cellular morphology, we performed MSC cultures on bidimensional glass coverslip (the Flat) and in our 3D Nichoid microstructured scaffolds [Fig. 1(a)]. To isolate the mechanotransduction effect of the Nichoid, we also assessed the extent to which pharmacologically treated cells can reduce the transmission of force to the nucleus. In bidimensional cell cultures, cells spread on the top of the glass coverslip, while in the three-dimensional condition, cells adhere to the scaffold in all spatial directions.

These different adhering conditions have significant repercussions on the cytoskeletal organization, as is evident from the actin fiber staining [Fig. 1(b_1_)]. The figure shows immunofluorescence acquisitions of actin cytoskeleton (green) and nuclei (red) for cells grown on Flat substrates, in 3D Nichoid, and cytochalasin-D (CD) treated cells. The CD treatment was applied to block the polymerization and consequently the elongation of actin filaments, thus reducing the cellular internal tension of spread cells.

To describe the spatial distribution of the actin stress fibers approaching the nuclear envelope, we investigated just the portion of the actin cytoskeleton that directly interacts with the cell nucleus [rectangular red ROI shown in Fig. 1(b_2_)]. Even though less efficiently than the pharmacological control, Nichoid-cultured cells show a reduced peak of actin fibers (Δ = 10%) reaching the nucleus, indicating a less polarized spatial distribution of actin cytoskeleton with respect to the spread cells on the Flat, suggesting a reduction in internal forces in this 3D culture condition. In addition to the reduction in the number of actin fibers reaching the nucleus, with the cytochalasin-D treatment, we also observed a randomization of their directionality, shown in the broadening of the fiber distribution curve [Fig. 1(b_2_)].

**FIG. 2.**
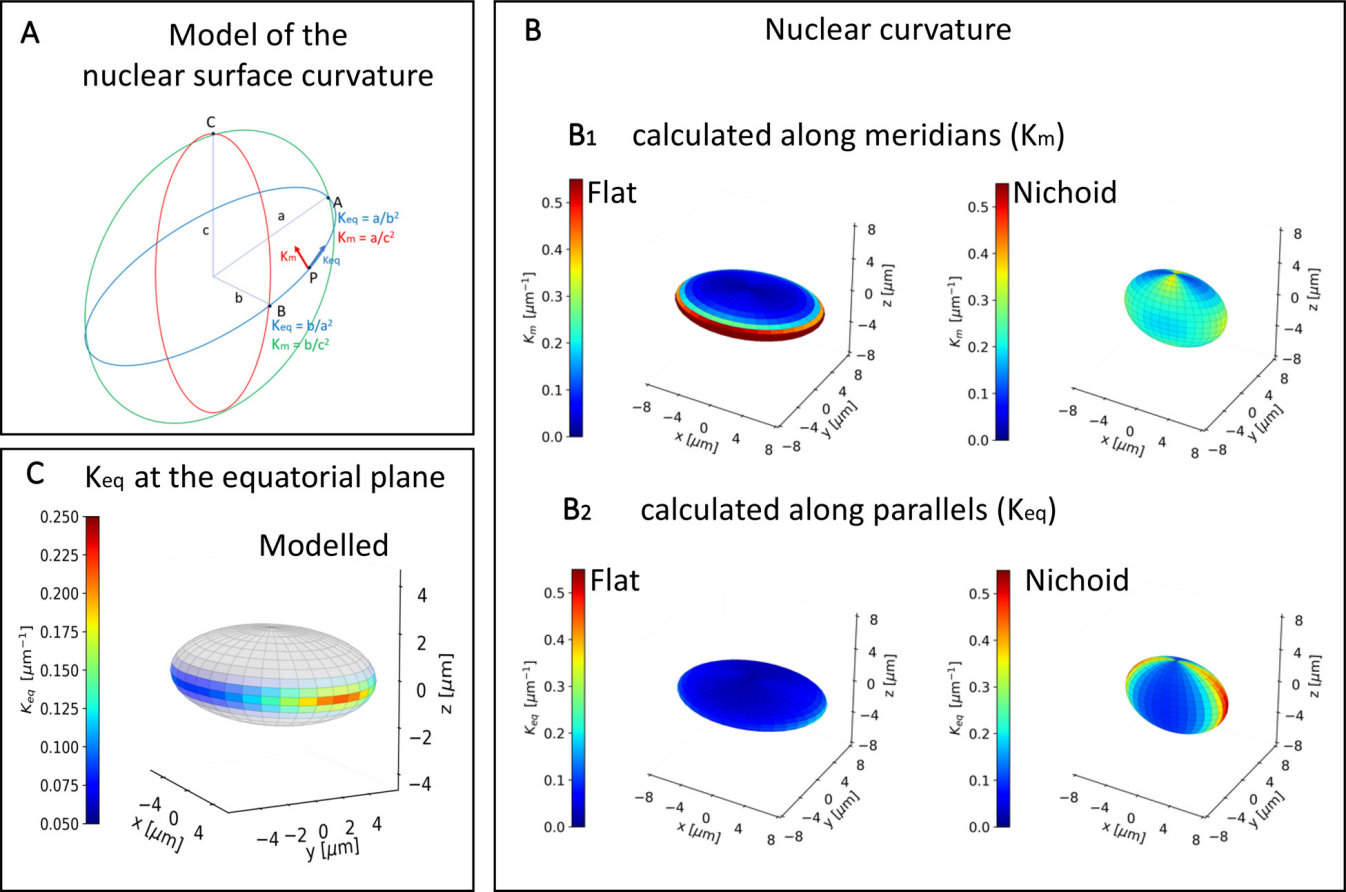
Model of the nuclear surface curvature. (a) Curvature analysis along meridians and parallels. (b) Nuclear curvature calculated along the meridians (b_1_) and parallels (b_2_). (c) Model of MSC nucleus spread on glass substrates in which the color map represents the nuclear curvature K_eq_, calculated at the nuclear equatorial plane for the Flat cell culture condition.

This difference in how the actin filaments are organized influences the nuclear shape, as indicated by the length of the semiaxes (a)–(c) reported in Fig. 1(c) and calculated by fluorescence images. Semiaxes (a) and (b) refer to the length of the major and minor semiaxes along the *xy*-plane, respectively, while c refers to the length of the semiaxes along the transverse direction. The cytochalasin-D treated 2D spread cells showed an intermediate nuclear geometry between cells on the Flat and in the Nichoid, where the shape of the nucleus is the most rounded. Indeed, progressing from the Flat, to the Flat + CD to the Nichoid cases, the length of the semiaxes in the *xy*-plane (a) and (b) decreases, while the length along the *z*-axis (c) increases. In fact, the ratio of the semiaxes length (b/c) is 3, 2, and 1, respectively, for the three cases listed above. In addition, the length c of the semiaxis along the *z*-direction in the Nichoid is twice as high as that obtained with the Flat sample (c_Nichoid_∼2c_Flat_).

These results indicate a nuclear shape moving from a scalene ellipsoid to a prolate ellipsoid. Figure 1(d) shows the modeled nuclear shape, integrated with the calculated curvature of the nuclear surface. The nuclear curvature K is defined as the root square of the Gaussian curvature (Bektas, 2017). As expected, Flat substrates are characterized by a marked Gaussian curvature, particularly along the equatorial plane, while the cells grown in the Nichoid show a more homogeneous curvature [Fig. 1(d)].

### B. Nuclear envelope permeability is affected by nuclear shape

To investigate the impact of nuclear shape on cell behavior, we investigated the relationship between the nuclear curvature and nuclear permeability. We analyzed the nuclear curvature according to the model presented in Fig. 2(a), uncoupling the curvature into its two fundamental components (K_m_ and K_eq_), which were calculated along the principal directions (meridians and parallels) [Fig. 2(b)]. Moving in the equatorial plane from point A, where the curvature K is maximum, to point B, where the curvature K is minimum, [see Fig. 2(a)], K_m_ varies by a factor of 1.3 for the Flat and 1.8 for the Nichoid, while K_eq_ varies approximately by a factor of 2 and 6 for the Flat and the Nichoid, respectively (see supplementary material, Table 1). This means that in both cell groups, the variation of K_m_ has less influence than K_eq_.

Given that the nuclear influx is more effective on the nuclear equatorial plane (Jacchetti *et al.*, 2021), we investigated the effect of the equatorial curvature on the translocation of molecules through the nucleus. Figure 2(c) highlights the variation in K_eq_ intensity along the equatorial plane in the condition of spread cells on Flat, showing a high permeability only in the polar zones of the cell nucleus.

**FIG. 3.**
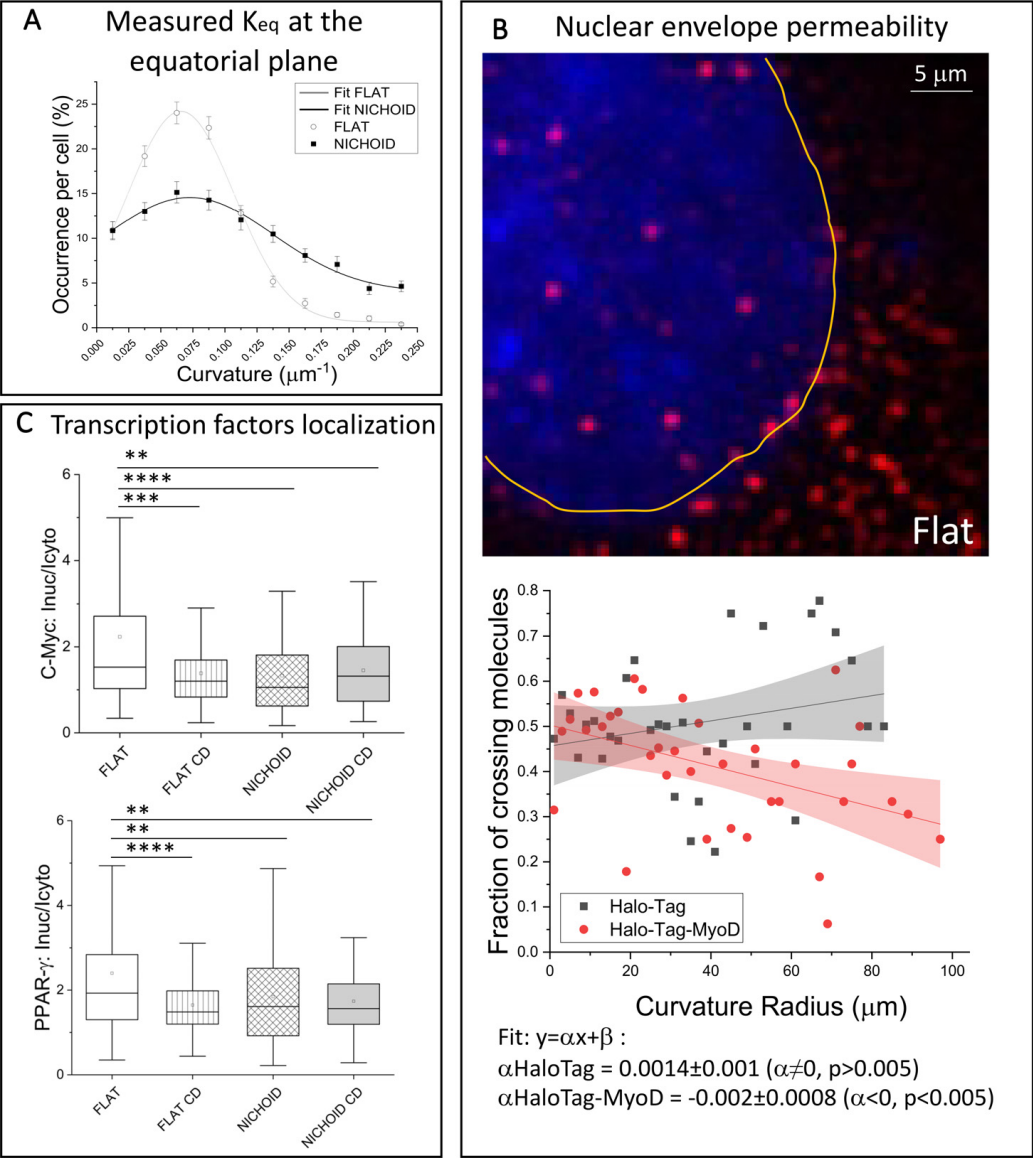
Nuclear envelope permeability regulates the import of transcription factors. (a) Frequency distribution of the nuclear curvature along the equatorial plane measured in cells grown both on the Flat and in the 3D Nichoid. White dots and black squares represent average data and standard error (235 and 120 cells were analyzed for the Flat and Nichoid cell culture conditions, respectively). Gray and black lines correspond to the Gaussian fits for the Flat and the Nichoid (b) A MSC transfected with HaloTag^®^ (red fluorescence). In blue, the cell nucleus; the yellow line highlights the nuclear envelope. The plot represents the fraction of molecules crossing the nuclear envelope as a function of curvature radius (R_eq_ = 1/K_eq_). Red dots and black squares represent frequency distribution data for facilitated diffusion (HaloTag^®^-MyoD) and passive diffusion (HaloTag^®^), respectively. Black and red lines are the linear fitting (y = αx + β) of dots and squares, respectively. Red and gray zones represent the 95% confidence level. The slope of the black line (α_HaloTag^®^_) is not statistically different from 0, meaning there is no dependence of permeability to the curvature radius Req. Instead, in the case of the MyoD protein (red line) α_HaloTag^®^-MyoD_<0 has a significance of p < 0.005, suggesting that with facilitated transport, the permeability of the nuclear envelope decreases with the curvature radius. In total, 30 000 HaloTag^®^-MyoD and 23 000 HaloTag^®^ molecules were analyzed in 20 cells, performed in three independent experiments. (c) Investigation of the nuclear level of two endogenous transcription factors: the more the nucleus is stretched, the more the proteins are localized in the nucleus. The effect of CD is similar to that of cell growth in the Nichoid. Statistical analyses were performed using the non-parametric Kruskal–Wallis test. ^**^, ^***^, and ^****^ correspond to p < 0.01, 0.001, and 0.0001, respectively.

Figure 3(a) shows the distribution frequency of the nuclear curvature K_eq_ of cells grown both on the Flat and in the Nichoid. The curvatures of the two nuclear configurations have the exact same range of values, but what changes is the number of times they occur. In both conditions, K_eq_ values can be fitted with a Gaussian distribution (fit-Flat and fit-Nichoid) with the peaks in the same range (0.05–0.075 *μ*m^−1^). The difference is the amplitude of the peak is more pronounced (1.7 times higher, 60% thinner) in the Flat sample, while the distribution related to the Nichoid widens at higher curvature values. This indicates a larger portion of the nucleus in which low curvatures are present in the case of spread cells.

We then performed single molecule tracking analysis to investigate nuclear permeability as a function of K_eq_. This technique, with our highly inclined and laminated optical sheet setup, is effective only at very short working distances and, therefore, can only be applied to MSCs spread on flat substrates. We transiently transfected cells with two different fluorescence labeled proteins: HaloTag^®^ (33 kDa), which is a small inert protein freely diffusing in cells, and HaloTag^®^ fused to the exogenous transcription factor MyoD (HaloTag^®^-MyoD). With a molecular weight of about 70 kDa, MyoD enters the nucleus by facilitated transport (Vandromme *et al.*, 1995). Figure 3(b) shows the detail of a cell transfected with HaloTag^®^ (red dots), where the nucleus (blue) is marked by a manually drawn ROI (yellow curve). From each time lapse (10 s), we measured the percentage of proteins crossing the nuclear envelope as a function of the nuclear radius of curvature K_eq_. The two proteins behaved differently in terms of their nuclear import (crossing ratio) as a function of the envelope geometry. Since the slope of the linear fit is not significantly different from 0, the passive diffusion of HaloTag^®^ across the nuclear envelope does not depend on K_eq_. On the other hand, the HaloTag^®^-MyoD nuclear influx increased with decreasing curvature radius, therefore the nuclear permeability is affected by the nuclear shape in terms of facilitated transport.

To highlight that the relationship between the nuclear permeability and the nuclear curvature is a generally applicable aspect, we used immunofluorescence microscopy to investigate the nuclear level of two endogenous transcription factors: PPAR-g and C-Myc. Figure 3(c) shows that the more the nucleus is stretched, the more the proteins are localized in the nucleus. The effect of CD is comparable to the cell expansion in the Nichoid.

### C. The Nichoid induces a homogeneous euchromatin distribution and affects gene regulation

**FIG. 4.**
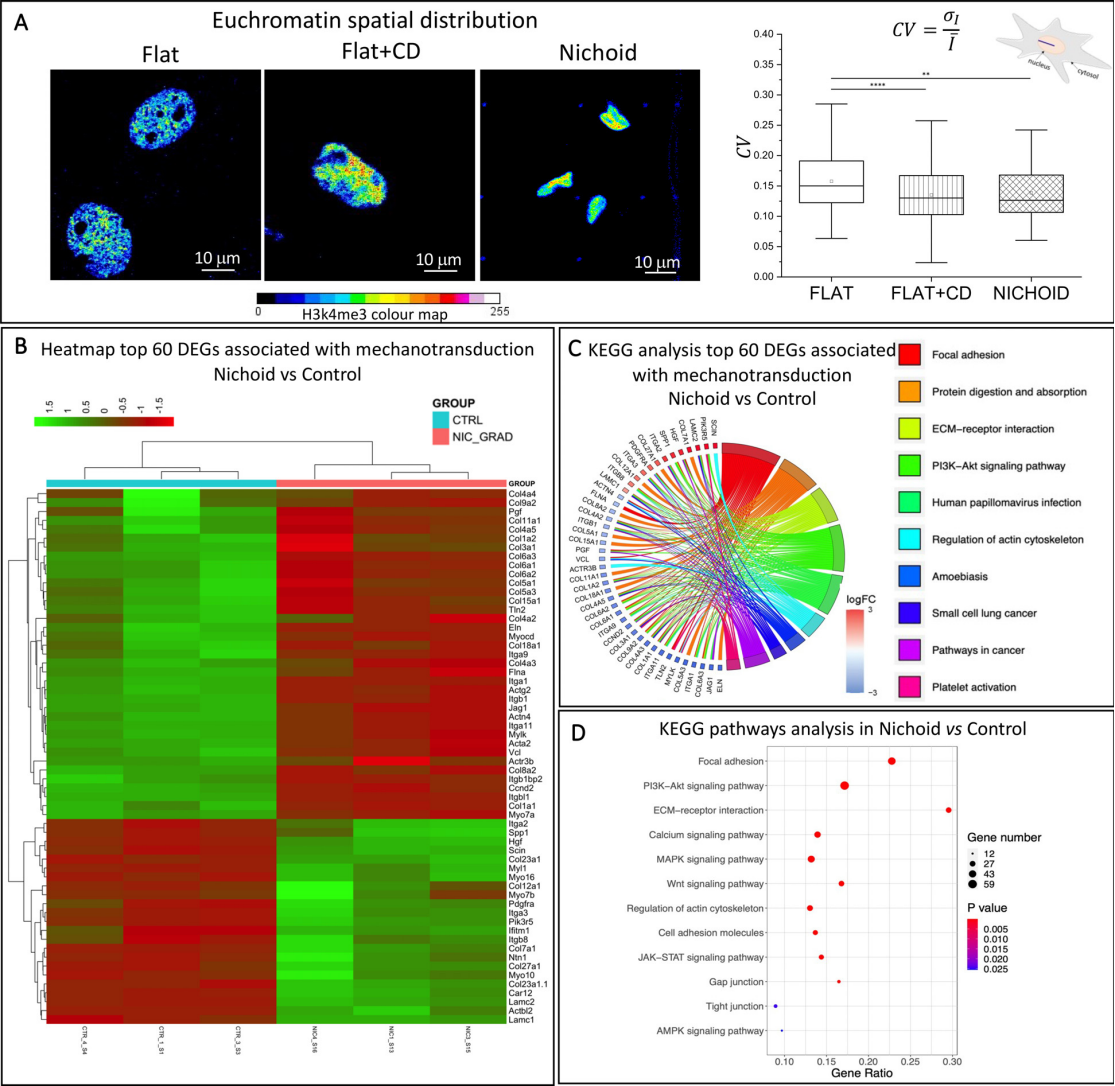
Euchromatin organization and gene expression in relation to mechanotransduction. (a) Color map of H3K4me3 fluorescence intensity of cells grown on the Flat, the Flat+CD, and in the Nichoid. Analysis of the coefficient of variation (CV) of H3K4me3 fluorescence intensity calculated on a linear ROI starting from the nuclear edge toward the inside of the nucleus. Results indicate that spatial distribution of active genes is more homogeneous in Nichoid-cultured cells. A total of 235, 193, and 120 cells for Flat, Flat+CD, and Nichoid samples, respectively, were acquired and analyzed in three independent experiments. Statistical significance was calculated using the non-parametric Kruskal–Wallis test. ^****^ corresponds to p < 0.0001. (b) Heatmap showing profile expression of 60 genes involved in mechanotransduction in MSCs cultured inside the Nichoid vs Flat substrates as control. The clustering analysis separates samples into two different “families.” Nichoid samples are reported in pink and Flat samples in light blue, respectively. (c) Top 10 significant pathways presented as GOChord plot. The panels were obtained considering the 60 mechanotransduction genes, and the color represents different pathways related to the deregulated genes. (d) KEGG pathways related to mechanotransduction of MSCs expanded inside the Nichoid compared with Flat substrate culture conditions. The y-axis represents the name of the pathway, the x-axis represents the gene ratio, dot size represents the number of different genes, and the color indicates the adjusted p-value.

To investigate the impact of culture conditions, and therefore the nuclear geometry, on nuclear mechanotransduction, we analyzed the euchromatin distribution and gene regulation. We first investigated the spatial distribution of the H3k4me3 fluorescence inside the nucleus [Fig. 4(a)] in Flat, Flat+CD, and Nichoid conditions. To compare the spatial distributions, we calculated the coefficient of variation (CV) of fluorescence intensity by drawing a line from the nuclear edge toward the nuclear center for each cell investigated [blue line in Fig. 4(a)]. In the graph, the Nichoid-cultured cells show a significantly lower CV, indicating a more homogeneous euchromatin distribution compared with the cells on Flat. When considering cells cultured on the 2D substrate treated with cytochalasin-D, the partial degradation of actin cytoskeleton induced a spatial redistribution of H3K4me3, which was similar to the one in the Nichoid. This result confirms that the euchromatin organization correlates with the transmission of intracellular forces.

To understand how much these events affect gene regulation, we performed a total RNA-sequencing analysis [Figs. 4(b)–4(d)]. Gene expression profiles of MSCs cultured inside the Nichoid differed significantly with respect to the Flat. Since the Nichoid affects the actin cytoskeleton and cell tension around the nucleus, we first focused on 60 genes related to mechanotransduction. As shown by the heatmap in Fig. 4(b), the analysis highlighted a significant dysregulation as different gene expression profiles can be visibly distinguished between the two conditions investigated.

To further investigate the role of these 60 mechanotransduction-related deregulated genes, we performed a functional enrichment analysis on a KEGG database via the g:Profiler web tool. The top 10 significant pathways are presented as a GOChord plot, which helps define the relation between the mechanotransduction genes and the pathways involved [Fig. 4(c)]. We found a deregulation of “focal adhesion,” “ECM-receptor interaction,” and “regulation of actin cytoskeleton” pathways when comparing MSCs cultured inside the Nichoid and on the Flat. In the top 10 pathways, we found that the “PI3K-Akt signaling pathway” was deregulated. This pathway helps regulate multiple cellular physiological processes such as cell cycle, cell growth, and proliferation (Borreguero-Muñoz *et al.*, 2019), as well as pathways related to cancer progression (Zhan *et al.*, 2017).

To confirm our previous observations concerning the impact of the Nichoid on mechanotransduction, we performed a KEGG pathways analysis to dissect the associated pathways [Fig. 4(d)]. We found four pathways implicated in the alteration of the membrane and cytoskeleton conformation (Focal adhesion, ECM-receptor interaction, regulation of actin cytoskeleton, and “cell adhesion molecules”), supporting the euchromatin morphological changes observed with confocal microscopy. Various kinase mechanotransduction-related pathways, such as “MAPK signaling pathway,” “PI3K-Akt signaling pathway,” and “AMPK signaling pathway” showed significant deregulation. Deregulated genes involved in these pathways control fundamental cellular processes including survival, apoptosis, proliferation, differentiation, and motility (Sun *et al.*, 2015). As expected, pathways related to cell organization such as “Gap junction” and “Tight junction” were deregulated, suggesting a different cell organization in the 3D microscaffold. “JAK-STAT pathway” and “Wnt signaling pathway” represent a rapid membrane-to-nucleus signaling module and are involved in fundamental cell processes such as stemness maintenance, cell migration, and proliferation (Hu *et al.*, 2021). The “calcium signaling pathway,” which is key to driving intracellular processes, was deregulated in MSCs inside the Nichoid with respect to the Flat substrate. Finally, by pathway analysis with gene ontology—biological processes—we also observed the deregulation of 19 pathways related to cell cycle progression and mitotic nuclear division (supplementary material, Fig. 1).

To confirm these observations, we performed a pre-ranked GSEA for GO cellular component, which returned 15 upregulated and 42 downregulated pathways. Interestingly, of these, the “nucleosome” and “DNA packaging complex” were upregulated (supplementary material, Table 2), highlighting that the chromatin in cells cultured inside the Nichoid are organized differently.

Using cytochalasin-D, we also assessed the relation between intracellular tension reduction and gene expression. RNA-seq analyses showed a significantly high number of deregulated genes between the three different conditions (#1843 Nichoid vs Flat, #847 Nichoid vs Flat+CD, and #74 Flat+CD vs Flat). As shown by the heatmap in the supplementary material, Fig. 2, Nichoid samples (light blue family) cluster together, and they are well separated from the bidimensional glass substrate sample (Flat, green family). In addition, treated samples (Flat+CD, the pink family) are perfectly positioned in the middle of the two reference samples. This strongly suggests that the reduction of the intracellular tension makes cells more similar to those cultured in the Nichoid, also when considering gene expression.

### D. Cells in Nichoid are characterized by low structured lamina mesh

**FIG. 5.**
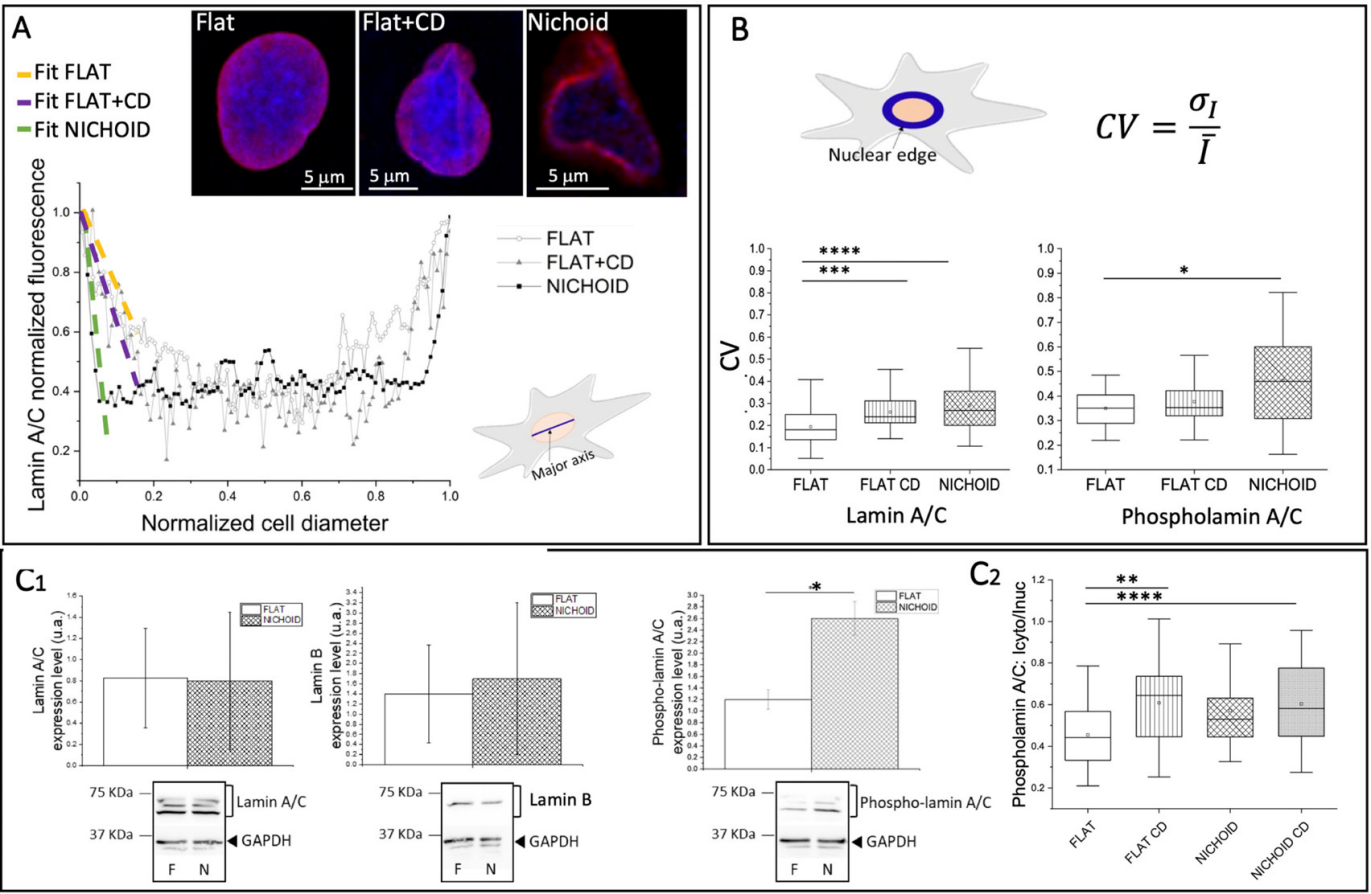

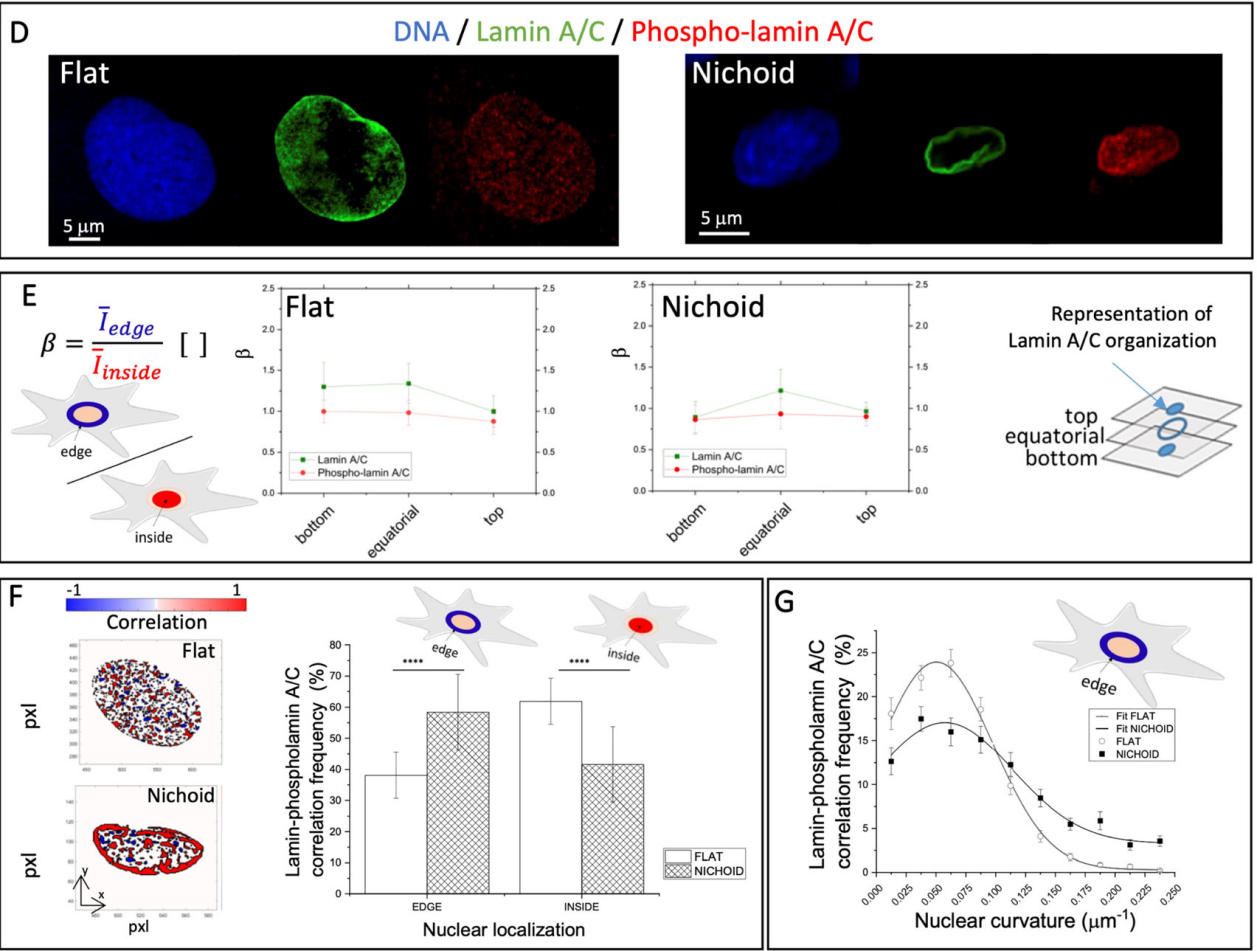
Lamins and phospho-lamin A/C expression and organization. (a) Images representing lamin A/C distribution (red) and DNA (blue) at the nuclear equatorial plane of cells grown on the Flat, the Flat+CD, and in the Nichoid substrates. The plot represents the lamin A/C fluorescence intensity along the nuclear major axis (blue line in the drawing of the cell) of one cell, for each growth condition investigated. White dots, gray triangles, and black squares represent Flat, Flat+CD, and Nichoid conditions, respectively. The slopes of relative linear fits (green, purple, and yellow lines) are used as index of the lamina thickness in the three cases. Treatment with cytochalasin-D makes the lamina thickness of cells grown on Flat more similar to those grown in the Nichoid. (b) Graphs of the coefficient of variation (CV) of the lamin A/C and phospho-lamin A/C fluorescence intensity, calculated along the nuclear edge (in blue in the drawing). In the Flat sample, the proteins, and in particular, lamin A/C, are more homogenously distributed than in the Nichoid, thus facilitating the formation of a highly structured mesh. The total number of analyzed cells for creating figures (a) and (b) is 58 and 55 for Flat and Nichoid conditions, respectively. Statistical analyses were performed using the non-parametric Kruskal–Wallis test. ^**^, ^***^, ^****^ correspond to p < 0.01, 0.001, and 0.0001, respectively. Three independent experiments were performed. (c) Investigation of the lamins expression level. (c_1_) Western blot analysis of lamin A/C, lamin B, and phospho-lamin A/C. Three independent samples of MSCs cultured on the Flat and in the Nichoid were analyzed (an example of western blot is reported in the supplementary material, Fig. 4). Statistical analyses were performed using the Mann Whitney test. Results show no significant variations in the expression level of lamin A/C and lamin B. The level of lamin A/C phosphorylated at serine 22 significantly increases in MSCs grown in Nichoid. (c_2_) Nuclear localization of Phospho-lamin A/C in different culture conditions shows That the nuclear levels of this protein increase in un-stressed culture conditions. In this experiment 56, 57, 55, and 49 cells were analyzed for the following conditions: Flat, Flat+CD, Nichoid, Nichoid+CD, respectively. Statistical analyses were performed using the non-parametric Kruskal–Wallis test. ^**^ and ^****^ correspond to p < 0.01 and 0.0001, respectively. (d) Representative images of cell nucleus (blue), lamin A/C (green), and phospho-lamin A/C (red) distribution acquired at the nuclear equatorial plane of MSCs grown on the Flat and in the Nichoid. the Calculation of the β coefficients at the bottom nuclear cap, at the equatorial one, and at the top nuclear cap (drawing on the right). β represents the relative intensity of lamin A/C mesh evaluated by comparing the nuclear edge fluorescence (blue ROI in the scheme) and the inner nuclear fluorescence (red ROI in the scheme). Unlike the cells grown in 3D, in the Flat samples, at the bottom cup level (i.e., close to the glass substrate), there is a reduced lamin A/C detection (β > 1). (f) Color maps of lamin/phosho-lamin A/C correlating (red) and anticorrelating (blue) pixels in two representative cells grown in the Nichoid and on Flat substrates. The plots represent the correlation frequency of lamin and phopho-lamin A/C evaluated at the equatorial plane in two distinct zones of the nucleus: the edge and its interior as represented by the blue and red ROIs in the cell drawings. The total number of analyzed cells for creating figures (e) and (f) is 58 and 55 for Flat and Nichoid conditions, respectively. Statistical analyses were performed using the non-parametric Mann–Whitney statistical test. ^****^ corresponds to p < 0.0001. Three independent experiments were performed. (g) Frequency distribution of the protein correlation as a function of the nuclear curvature (blue ROI in the drawing of the cell). White dots and black squares represent average and standard error, and gray and black lines correspond to Gaussian fits for the Flat and the Nichoid, respectively. The total number of analyzed cells is 58 and 55 for the Flat and the Nichoid, respectively. Statistical analyses were performed using the Mann–Whitney test. Three independent experiments were performed.

Since the nucleoskeleton plays an important role in mechanotransduction, we investigated the lamina organization by immunofluorescence assay and lamin expression levels by western blot assay. First, we examined the effect of the Nichoid culture condition on the lamin A/C organization. We measured lamin A/C fluorescence intensity at the equatorial plane along the nuclear major axis [blue ROI in the cell in Fig. 5(a)] in Flat and Nichoid conditions. The fluorescence intensity indicates the level of lamina mesh distribution and its spread toward the nuclear center (Stiekema *et al.*, 2021). In cells cultured in Nichoid, fluorescence intensity sharply decreases from the nuclear edge to the center. Instead, in cells spread on Flat, the lamin A/C fluorescence had a more homogenous distribution extending toward the nuclear interior areas. We then performed a linear fit of the data and calculated the slope ( α) for each cell. This slope represents the lamin A/C mesh spreading toward the nuclear center. Figure 5(a) reports an example of the analyses and shows one representative fluorescence lamin A/C profile for each investigated growth condition (Flat, Flat+CD, and Nichoid). To compare all curves, the intensity profile values (*I_i_*) were normalized to the maximum intensity value I_max_ of the related dataset, and the cell diameter was normalized to 1. Our results show that at the equatorial plane, the lamin A/C thickness occupied the nuclear portion equal to 21% in cells adhering to the Nichoid, and 64% in cells spread on the Flat substrate.

To confirm the hypothesis that the lamina rearrangement is induced by changes in the internal cellular tension, we also considered MSCs on the Flat+CD. The disruption of the actin cytoskeleton reduces the thickness in the lamin A/C, as highlighted by the average slope, calculated for the three culture conditions ( $\alpha_{\mathrm{Flat}}$= −0.183 ± 0.089, $\alpha_{\mathrm{Flat}+CD}=$−0.386 ± 0.214, and $\alpha_{\mathrm{Nichoid}}=$−0.765 ± 0.356). In fact, the fit slope increases as the intracellular tensions decrease. As shown in the supplementary material, Fig. 3(a), the cytochalasin treatment does not impact the organization of the lamina in the Nichoid culture.

Still at the cellular equatorial plane, we then characterized the distribution of lamin A/C and phospho-lamin A/C along the nuclear edge [the blue ROI in Fig. 5(b)]. We calculated the intensity coefficient of variation parameter [CV, Eq. (2) in Sec. V] to describe the distribution homogeneity of the two proteins.

The results reported in Fig. 5(b) and the supplementary material, Figs. 3(b) and 3(c), show that cells grown on the Flat are characterized by a more homogeneous distribution of both proteins than cells in the Nichoid (CV_Nichoid_ > CV_Flat_). Treatment with cytochalasin-D only affects the lamin A/C distribution of spread cells (CV_Nichoid_ ∼ CV_NichoidCD_ ∼ CV_FlatCD_), and it only enhances the homogeneous distribution of phospho-lamin A/C when cells are cultured in the Nichoid. These results demonstrate that cellular tension mainly influences the organization of the nuclear structural proteins (i.e*.,* lamin A/C) and affects the dispersion of free diffusive proteins (i.e*.,* phospho-lamin A/C).

To further investigate whether the different culture conditions (Flat and Nichoid) might influence the expression levels of lamins, we performed western blot analyses [Fig. 5(c) and supplementary material, Fig. 4]. The expression of the lamins [Fig. 5(c_1_)] involved in the lamina mesh formation (i.e., lamin A/C and for comparison also lamin B proteins) did not exhibit specific variations. Instead, in the case of phospho-lamin A/C, which plays a role in lamina renewal and gene regulation (Kochin *et al.,* 2014), we observed that the expression of lamin A/C phosphorylated at serine 22 (named here phospho-lamin A/C) was more pronounced in cells grown in the Nichoid. We thus used immunofluorescence microscopy to investigate whether phospho-lamin A/C expression levels vary between the nucleus and cytoplasm [Fig. 5(c_2_)]. Our results show that the nuclear levels of this protein only decreased significantly in the CD treated conditions. The 3D scaffold thus only seems to influence the overall level of phospho-lamin A/C but not its distribution in the cell.

To better analyze the organization of the proteins in the cell nucleus, we carried out further immunofluorescence analyses. Figure 5(d) shows the spatial distribution of the two states of the lamin A/C protein, highlighting that both proteins are distributed differently in Flat or Nichoid conditions. For example, the phospho-lamin A/C seems to be more clusterized in the nuclear center in the Flat sample.

This evidence prompted us to investigate the organization of nuclear lamina at the two nuclear caps, and at the top and the bottom of the cell [Fig. 5(e)]. For each plane (bottom, equatorial, top) we calculated the parameter β [Eq. (3)], defined as the average fluorescent intensity of the nuclear edge [blue ROI, Fig. 5(e)] over the average fluorescent intensity of the inner part of the ROI [red ROI, Fig. 5(e)]. The plots show that phospho-lamin A/C does not have a preferential localization (β∼1) in none of the analyzed nuclear planes either in cells cultured on Flat or in 3D Nichoid samples. In contrast, lamin A/C is distributed differently; in MSCs spread on Flat, lamin A/C at the bottom and equatorial planes is primarily located at the nuclear edge (β > 1) and shows a uniform distribution (β ∼ 1) at the top cap of the nucleus. Instead, in cells grown in the Nichoid, lamin A/C showed a homogeneous distribution at the bottom and the top nuclear caps (β ∼ 1), while at the equatorial plane, it is mainly located close to the nuclear edge (β > 1). These results are further supported by the images reported in the supplementary material, Fig. 5, showing the *z-*stack images of MSCs grown on the Flat and in 3D Nichoid. The red rectangles indicate the images acquired at the nuclear equatorial plane, the yellow and green ones correspond to 2 *μ*m thick slices acquired at the bottom and top of the nucleus, where it is evident that the lamin A/C mesh is not visible in the bottom cap (i.e., yellow rectangle) of the cells spread on the flat substrate. To better understand the reciprocal spatial organization of the lamin A/C and the phospho-lamin A/C, we investigated the regions characterized by the co-presence of the two proteins. Spatial correlation analysis is shown in Fig. 5(f) (red = correlation and blue = anti-correlation). The results show that around 60% of the correlating pixels are located in the inner part of the nucleus in cells cultured on Flat, and at the nuclear edge in the Nichoid culture condition.

Finally, at the nuclear edge, we also investigated the colocalization level as function of the nuclear curvature [Fig. 5(g)]. The graph shows that in both cell populations (i.e., on Flat and in the Nichoid), the colocalization mainly occurred for small nuclear curvatures. On Flat, the fitting curve was slightly shifted to the left (the maximum corresponded to nuclear curvatures of 0.05 vs 0.057 *μ*m^−1^ for the Flat and the Nichoid, respectively), with higher Gaussian indexes (Peak_height_Flat_ = 24%, FWHM_Flat _= 0.095 ± 0.004 *μ*m^−1^ vs Peak_height_Nichoid_ = 17%, FWHM_Nichoid_ = 0.114 ± 0.012 *μ*m^−1^, where FWHM means full width at half maximum).

### E. Lamin A/C organization in complex structure corresponds to higher dampen ability

**FIG. 6.**
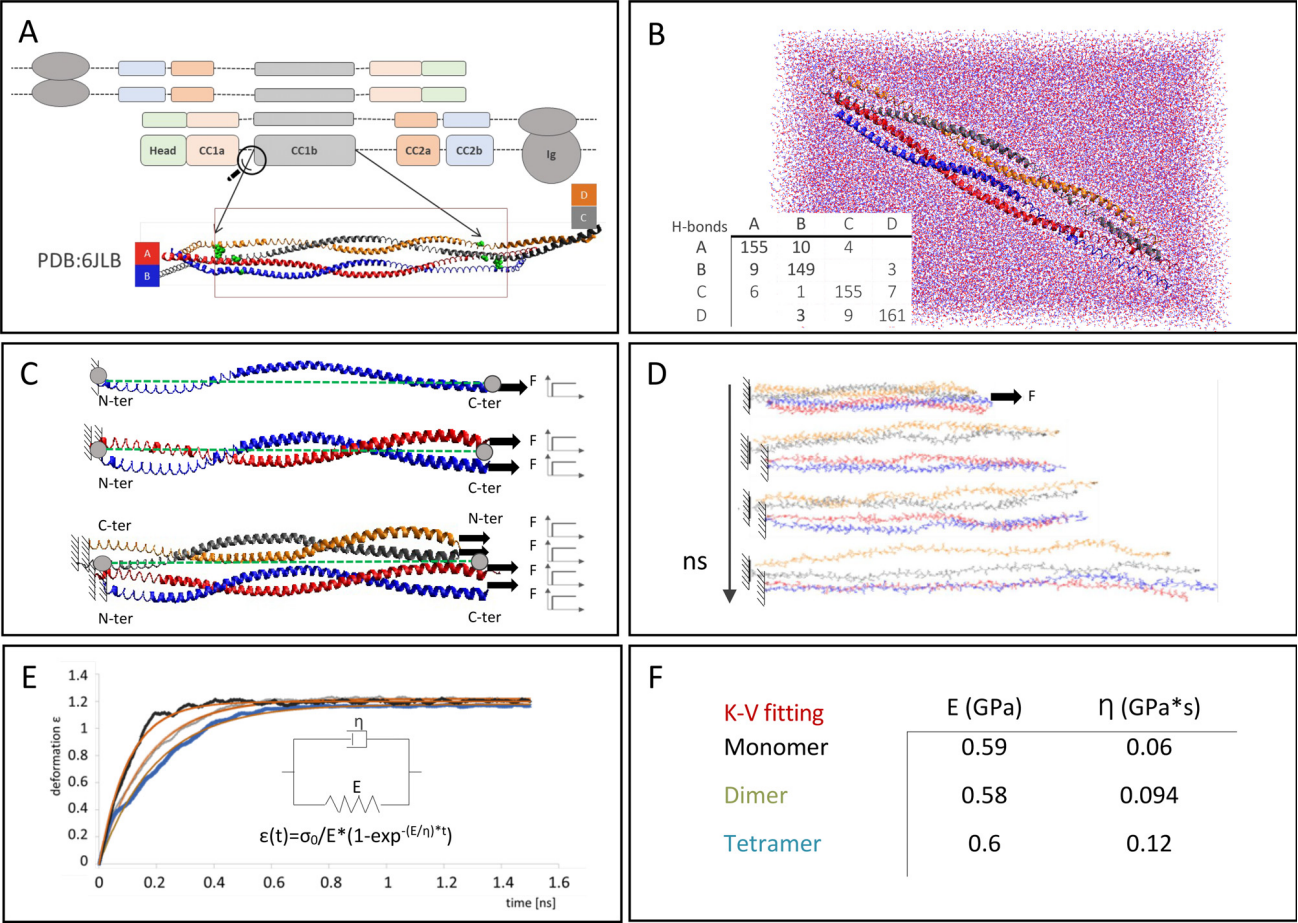
Computational prediction of lamin A/C deformation. (a) The lamin A/C tetramer configuration, consisting of two antiparallel dimers mainly characterized by the coiled-coil domains (CC1a, CC1b, CC2a, and CC2b) represented by colored rectangles. (A-Inset) VMD cartoon of the structure of CC1b domain, extracted from tetramer lamin A/C crystal structure (PDB code: 6JLB). (b) The CC1b domain solvated in water box and equilibrated for 600 ns. The H-bond numbers in the chains are reported in the table. (c) The creep test: a step force F was applied to one chain terminal (C-ter for chains A and B; N-ter for chains C and D). The other terminal of each chain was fixed. Monomer, dimer, and tetramer were reduced to an equivalent single chain (green dotted lines) linking the average coordinates (gray spots) of fixed atoms and the average coordinates of steered atoms. (d) Time evolution of lamin A/C tetramer elongation during the creep simulation. (e) Monomer (gray), dimer (green,) and tetramer (light blue) equivalent-chain elongation curves during the simulation. Red curves represent Kelvin–Voigt fitting for each configuration. Kelvin–Voigt model with the related equation, consisting of spring in parallel to dash-pot element. (f) Young's modulus and viscosity parameters extracted from Kelvin–Voigt equation for each configuration. While Young's modulus is almost constant for all the configurations, the viscosity term increases with structure complexity.

To predict the effects of the lamin superstructure and its role in nuclear protection, we investigated the mechanical properties of lamin A/C. We investigated the lamin tensile stress response because this is the most likely kind of stress to be transmitted by the LINC complex to single lamin filaments assembled in the structured mesh at the nuclear level. Intermediate filaments are mainly responsible for resisting tensile loads by reinforcing the regions of the cell and nuclear membranes that are subject to stress. We used molecular dynamic simulations and isolated the contribution of monomer, dimer, and tetramer arrangements. Due to the highly repetitive lamin structure, which mainly consists of coiled-coil domains, we investigated the mechanical features of lamin A/C coiled-coil domain 1b (CC1b) [Fig. 6(a)].

The CC1b tetramer model was solvated in a water box [Fig. 6(b)] to perform molecular dynamic simulations. The root mean square deviation (RMSD) curve calculated on the last 70 ns of the equilibration simulation (RMSD= 2 ± 0.5 Å) confirmed the achievement of a stable tetramer configuration. We then estimated the H-bonds occurring in the tetramer configuration. The inset in Fig. 6(b) reports the number of intra- and inter-chain H-bonds with a more than 20% occurrence in the last 70 ns of equilibration. Intra-chain bonds are almost constant of the four monomeric chains and represent most of the H-bonds.

*In silico* creep tests were performed on monomer, dimer, and tetramer arrangements [Fig. 6(c)] until total protein distention was reached [Fig. 6(d)]. Structure deformation over time revealed an exponential trend typical of viscoelastic material, characterized by Young's modulus (E) and the viscosity parameter (η) [Fig. 6(e)] (Gautieri *et al.*) Fitting the elongation curve by the Kelvin–Voigt model, Young's modulus and viscosity parameters were extracted for each level of aggregation reported in Fig. 6(f). While Young's modulus was almost constant among the various aggregation levels (about 0.23 GPa for monomer, dimer, and tetramer), the viscosity predicted value increases from the monomer to the tetramer arrangement, corresponding to 0.06, 0.094, and 0.12 GPa s for the monomer, dimer, and tetramer, respectively. Considering the constant intra-chain H-bonds in the four monomeric chains, the viscosity variation correlated with inter-chain H-bonds occurring within the dimer and the tetrameric arrangements.

## III. DISCUSSION

The high sensitivity of MSCs to physical stimuli from the extracellular environment represents a new target for controlling their fate and functionality (Discher *et al.*, 2009; Maul *et al.*, 2011; Delaine-Smith and Reilly, 2012; Tsimbouri *et al.*, 2014; and Anderson *et al.*, 2016). Through a combination of computational and experimental analyses, we characterized the main actors of the mechanotransduction pathway triggered by the Nichoid architecture compared to the standard glass culture substrate (the Flat), as summarized in Table 3 of the supplementary material.

Unfortunately, technological limitations prevented us from using a 2D flat substrate produced by the two-photon polymerization lithographic technique, which would be perfect for isolating the contribution of the scaffold architecture from the chemical and mechanical one (Ricci *et al.*, 2017; Conci *et al.*, 2022). First, even by creating a flat substrate composed of a thin monolayer of photoresist, the sample produced would have had different physicochemical properties to the Nichoid, without additional benefits over the glass. In addition, the high auto-fluorescence of the photoresist in the range of visible light would prevent a fluorescence microscopy investigation.

To validate the usability of the glass coverslip as a bidimensional control sample in terms of substrate stiffness, we performed finite element simulations showing that despite the lower Nichoid stiffness compared to the glass, the 3D scaffold was still non-deformable (less than 15 nm applying a 10nN force per adhesion point) when subjected to forces that cells are able to exert (data not shown). This result supports the use of the glass coverslip as a reference bidimensional substrate, introducing the 3D architecture as the main element of differentiation between the two conditions.

The 3D architecture of the scaffold influences the rearrangement of the cellular adhesion process by triggering the cytoskeleton reorganization (Zonderland *et al.*, 2020) and affects the intracellular tension, which then impacts on gene expression (Smith *et al.,* 2022; Balaban *et al.*, 2021; and Fu *et al.*, 2010). To confirm this, we investigated different aspects of the mechanotransduction process induced by the Nichoid ranging from the actin fiber distribution around the nucleus, the nuclear shape, molecular nuclear trafficking, euchromatin spatial distribution, gene expression profile, and the lamin arrangement.

To corroborate the hypothesis that in the Nichoid, intracellular tension is the engine that modulates the cell phenotype, we introduced an additional experimental control consisting of cells treated with the drug, cytochalasin-D (CD). Our experiment showed that the drug only affects spread cells cultured on flat samples (Flat+CD), while there is no significant effect on cells cultured in the Nichoid in terms of actin organization (data not shown). By destroying the actin cytoskeleton and thus the intracellular tension, cytochalasin-D represents the key to interpreting the results obtained in the Flat and the Nichoid samples.

In terms of cytoskeletal organization, the Flat exhibited numerous thick actin fibers crossing the whole cell and inducing high intracellular tension. In contrast, actin fibers in the Nichoid were mainly located at the cell periphery, with a 10% reduction in oriented actin fibers approaching the nucleus [Fig. 1(b_2_)]. The cytochalasin-D treatment in the Flat sample induced a partial disruption of the cytoskeletal organization, confirming the Flat+CD as a good sample reference in which intracellular forces are reduced.

The different distribution of actin fibers induces a different adaptation of the nuclear shape (Khatau *et al.*, 2009; Vishavkarma *et al.* 2014; and Davidson and Cadot, 2021). Similarly, while in the Flat, the actin fibers approaching the nucleus are associated with an elongated nuclear shape; in the Nichoid, the reduction in actin fibers corresponds to a more roundish nuclear shape. The reduction in the intracellular tension via the chemical treatment produced a nuclear profile that was in between the Nichoid and Flat conditions, thus supporting the fact that the Nichoid modulates the force transmission to the nucleus [Figs. 1(c) and 1(d)].

Based on the different nuclear morphologies induced by the specific supports, we then investigated whether nuclear curvature could be associated with the Nichoid-related gene regulation observed in Fig. 4 and in previous studies (Remuzzi *et al.*, 2020; Testa, 2023). First, we observed differences in terms of chromatin distribution (Reynolds *et al.*, 2021; Damodaran *et al.*, 2018; Briand and Collas, 2020; and Cremer and Cremer, 2001). The MSCs cultured in the Nichoid showed a more homogeneous spatial distribution of euchromatin compared to the Flat controls, suggesting a higher accessibility to the active gene regions. In line with this, Heo and colleagues observed a reduced H3K4me3 clustered distribution in MSCs grown on soft compared to stiff substrates (Heo *et al.*, 2020). Considering that MSCs in the Nichoid maintain higher levels of stemness (Remuzzi *et al.*, 2020), our results are also supported by Ricci *et al.*'s experiments with stochastic optical reconstruction microscopy (STORM), which showed that the distribution of nucleosome nanodomains is more homogeneous in pluripotent stem cells compared to somatic cells (Ricci *et al.*, 2015).

As a complementary aspect of the chromatin distribution, and thus DNA accessibility to transcription factors, we also investigated nuclear permeability. In a previous study, we demonstrated that molecular transport in the nucleus occurs predominantly at the equatorial plane, and that molecular nuclear trafficking is globally higher in spread cell conditions than in the 3D Nichoid, especially for proteins with a high molecular weight (Jacchetti *et al.*, 2021). Here, we quantitatively estimated the amount of HaloTag^®^ and HaloTag^®^-MyoD proteins entering the cell nucleus as a function of its equatorial curvature radius (K_eq_^−1^).

We used MyoD as the transcription factor tester because (i) it is not spontaneously expressed by MSCs at early passages, (ii) it flows into the nucleus by facilitated translocation, (iii) it is a small protein (∼40 kDa), which should facilitate the transfection efficiency. For the analysis, we used highly inclined laminated optical (HILO) sheet microscopy, which works well for very small working distances (such as for the Flat) (Liu *et al.*, 2016; Swinstead *et al.,* 2016; Morisaki *et al.*, 2014; Mazza *et al.*, 2012; Mueller *et al.*, 2010; and Li *et al.*, 2019) but unfortunately prevented us from applying the same analysis to the Nichoid. In our analyses, we observed that around 50% of the molecules approaching the peri-equatorial zone of the nuclear envelope passed through it. However, while passive diffusion (i.e*.,* HaloTag^®^ molecule) is not affected by nuclear curvature, in facilitated transport (i.e*.,* HaloTag^®^-MyoD protein) the analysis of protein translocation probability suggests that there is a relationship between translocation and the nuclear curvature: a smaller nuclear curvature radius corresponds to a higher accessibility of transcription factors [Fig. 3(b)].

Nuclear curvature thus needs to be taken into account, together with other factors contributing to nuclear remodeling and permeability, such as nuclear cargoes (Paci *et al.*, 2021; Sun *et al.*, 2008; Lowe *et al.*, 2015; and Lyman *et al.*, 2002), calcium levels (Itano *et al.*, 2003; Malviya and Rogue, 1998), and enzymes (Lomakin *et al.*, 2020; Venturini *et al.*, 2020; Sales Gil *et al.*, 2018; and Kosako and Imamoto, 2010). To corroborate our data, we tested the nuclear level of two endogenous transcription factors in the different culture conditions. As shown in Fig. 3(c), our results highlight that the more stretched nuclei are more permeable. In fact, the nuclear level of two transcription factors decreases when cells are cultured in the Nichoid or when treated with cytochalasin-D. Similar results were also obtained using puromycin (supplementary material, Fig. 6), suggesting that the reduced intracellular tension has a similar effect to the transport inhibition on TF nuclear internalization.

The different nuclear trafficking and specific euchromatin distribution induced by 3D Nichoid suggested different gene transcription activities as confirmed by RNA-seq analysis. In line with Rey *et al.*, our results showed that cell expansion inside the 3D Nichoid influences different pathways in relation to calcium homeostasis and cellular quiescence, proliferation, stemness, longevity, and, most of all, pathways related to mechanotransduction (Rey *et al.*, 2020). This supports the hypothesis that the Nichoid acts on cell behavior by modulating force transmission to the nucleus. In addition, our results show that the treated samples (Flat+CD, the pink family) were perfectly positioned in between the Flat and the Nichoid, confirming the reduction in the intracellular tension as a distinctive key of the Nichoid structure (supplementary material, Fig. 2).

In line with the literature showing substrate-dependent lamina organization (Cosgrove *et al.*, 2021; Greiner *et al.,* 2015), our two culture conditions were characterized by different arrangements of lamins and expression. In agreement with Swift and colleagues, our results showed an equal expression of lamin B (Swift *et al.*, 2013). We thus focused on lamins A/C as the literature also indicates that they, and not lamin-B, regulate nuclear mechanics (Lammerding *et al.*, 2006). In addition unlike lamina B, the detachment of lamin A/C from the nuclear membrane only occurs when there is rapid or extreme mechanical stress (Pfeifer *et al.*, 2022).

Lamin A/C formed a thin layer close to the whole nucleus in the 3D Nichoid, while spreading toward the nucleus in Flat samples [Fig. 5(a)]. The results obtained using CD-drug corroborate the idea that it is the internal tension of the cell (i.e., incoming force to the nucleus) that causes this rearrangement of the lamina toward the nucleus [Fig. 5(a), supplementary material, Fig. 3(a)].

We also observed that the Nichoid is characterized by a high variation in lamin A/C fluorescence intensity compared to the other samples [CV_Nichoid_ ∼ CV_Nichoid+CD_ ∼ CV_Flat+CD_ > CV_Flat_, Fig. 5(b), supplementary material, Fig. 3(b)], and that in this culture condition, the phospho- and lamin A/C mainly co-localize at the edge of the cell nucleus [Fig. 5(f)].

Together with the high expression of phospho-lamin A/C in cells grown in the Nichoid [Fig. 5(c_1_)], and in agreement with the study by Kochin *et al.* (2014) and Machowska *et al.* (2015), these results suggest a dynamic active turnover between the two forms of protein in the Nichoid. In contrast, in spread cells, the reduced presence of phospho-lamin A/C is consistent with the hypothesis of a well-structured lamina mesh. Further information on the state of turnover of the lamina can be deduced from Fig. 5(g), which, in agreement with Pfeifer and colleagues (Pfeifer *et al.*, 2022), shows that the correlation between lamin and phospholamin A/C is greater in conditions of low curvature.

The remodulation of the lamina mesh in the Nichoid is an indication of the limited need to form a protective shell, as suggested by Ihalainen *et al.* (2015). To confirm the correlation between a highly structured lamina and the protection provided by the nuclear structure, we performed a molecular dynamic (MD) analysis using the lamin A/C coiled-coil domain 1b (CC1b) [Fig. 6(a)] as representative of the whole protein behavior [Fig. 6(e)] (Ackbarow and Buehler, 2007; Gautieri *et al.*, 2012). This revealed that Young's modulus (0.6 GPa) and viscosity parameter, which ranged from 0.12 to 0.06 GPa s, were in line with the literature (Schäpe, *et al.*, 2009; Guzmán *et al.*, 2006; Qin *et al.*, 2009; Laurini *et al.*, 2018; Zhang *et al.*, 2008; and Qin *et al.*, 2009).

The viscosity increased with the structure's hierarchical complexity, likely due to the increasing number of inter-chains H-bonds (i.e*.,* intra-dimer and inter-dimer). This supports the hypothesis that the highly structured lamin A/C experimentally observed on the Flat corresponds to a high ability to dampen external forces and thus protect the nuclear structure from extra cellular matrix modifications. In contrast, the de-structured lamina in the Nichoid suggests a reduced damping need, thus supporting the hypothesis of the reduced force exerted by the 3D Nichoid. Similar results, performed on cells grown on soft substrates, have revealed a less structured lamina network without intense cytoskeletal input forces (Qin and Buehler, 2011; Swift *et al.* 2013; Wren *et al.* 2012; and Buxboim *et al.*, 2014). In 2014, Buxboim and colleagues demonstrated that the fraction of phospho-lamin A/C decreases concomitantly with greater nuclear spreading, corresponding to cells grown on a stiff scaffold or generally to cells with a high internal tension.

Another key aspect is how lamin A/C and its post transcriptional modification affect gene activity. The lamina mesh plays an important role in binding heterochromatin and pushing it close to the nuclear envelope. However, the phosphorylated state of lamin A/C also has important functions, for example, in binding genes so that they remain active, as suggested by Naetar *et al.*(2021), Naetar *et al.* (2017), Machowska *et al.* (2015), Ikegami *et al.* (2020), Manzo and van Steensel (2020), and Gesson *et al.* (2016).

In our study, we observed that phospho-lamin A/C proteins are highly expressed in cells grown in the Nichoid and that its intranuclear distribution is homogeneous. We also found a cytosol-nuclear ratio imbalance toward the cytosol compartment in the case of reduced intracellular tension [Fig. 5(c_2_)]. When considering cells spread over the flat samples, the phospho-lamin A/C level is reduced, and a clustered distribution is observed in the nucleus, where the genes that need to be kept active are located [Figs. 5(c)–5(e)]. Future experiments will be performed to demonstrate this relationship, however in the meantime, these differences in lamina organization may contribute to massive gene deregulation highlighted by RNA-seq analysis (1843 deregulated genes between the Nichoid and Flat samples).

## IV. CONCLUSIONS

With the long-term aim of regarding the Nichoid as a tool for preserving the MSC phenotype and using it in clinical applications, in this paper, we have extensively investigated the main mechanoresponsive elements of cells in order to understand the relationship between the MSC culture in the Nichoid and gene expression. Comparing the Nichoid with the Flat culture conditions, we observed that the physical stimuli generated by the Nichoid's 3D geometry reduced the number of actin fibers approaching the nucleus. The nucleus was thus maintained in a roundish shape, and the lamina mesh did not thicken, a necessary condition to protect the cell nucleus from external stimuli and to regulate gene activation. In this scenario, in fact, MSCs maintain a homogeneous distribution of euchromatin and an altered nuclear trafficking of transcription factors, which affects the transcriptional activity.

We have revealed some key aspects of the Nichoid's mechanism of action on stem cells, which were shown to be based primarily on reducing force transmission to the nucleus. We believe that our results represent a step forward in the mechanical control of stem cells and pave the way to new strategies for their translation to clinical use.

We now plan to investigate the effects of impaired lamina distribution on gene transcription and how this may correlate with pathological conditions such as laminopathies, cancer, and neurodegeneration.

## V. METHODS

### A. Scaffold microfabrication

The 3D microstructured scaffolds used in this work, and referred to here as the Nichoid, were produced with a highly spatially resolved two photon polymerization technique in the commercially available SZ2080 photoresist, composed of a sol-gel-synthetized silicon (S)-zirconium (Z) hybrid inorganic–organic resin, described in detail by Ovsianikov *et al.* (2012).

The Nichoid (MOAB Srl, Milan, Italy) is made up of graded pores (10/20/30 × 10/20/30 × 15 *μ*m^3^), which are combined to create a unique matrix with a volume of 450 × 450 × 30 *μ*m^3^. It was directly fabricated on top of standard culture substrates such as the Nunc Lab-Tek-8-chamber wells (Thermo Fisher Scientific, Italy) and glass culture coverslip (1.2 cm in diameter), which are both suitable for fluorescent microscopy. In the first substrate, the microstructures were fabricated in the four central wells (7 × 9 mm^2^ each) and spatially organized in 2 × 2 units. In the latter, a circular area with a diameter of 8 mm was fully covered with Nichoids [as shown in Fig. 1(a)]. Details on how the Nichoid is manufactured are reported in Ricci *et al.* (2017).

In all experiments, the samples underwent the same treatment steps, except for exposure to the polymerization laser beam, which was obviously not used for the Flat control samples.

### B. Cell culture substrate preparation

Throughout this work, we compared two cell populations: spread cells vs roundish cells, which were obtained by seeding the MSCs on the two different substrates: glass substrates (the Flat) and the 3D engineered microstructured (the Nichoid). Before use, each substrate was sterilized with ethanol 70% and irradiated by UV light for at least 10 min. Ethanol was then removed, and the structures were washed twice with sterile water. Before cell seeding, substrates were dried out. No other pretreated condition was used.

### C. Cell seeding protocol

Rat bone marrow MSCs provided by IRCCS-Istituto di Ricerche Farmacologiche “Mario Negri” (Bergamo, Italy) were isolated from adult rat bone marrow. The isolation protocol is described in Zoja *et al.* (2012) and summarized therein. The MSC stock was derived from a pool of MSCs obtained from bone marrow collected from 12 Lewis rats. Briefly, bone marrow was flushed from the shaft of the bone and filtered through a 100-*μ*m sterile filter. Filtered bone marrow cells were plated in α-MEM supplemented with 20% FCS and penicillin-streptomycin, and were left to adhere for 24 h. After 2–3 weeks, sub-confluent cells were detached by trypsin-EDTA and harvested in FBS + 10% DMSO. FACS analysis revealed that MSCs were negative (98% negative cells) for the hematopoietic marker CD45 (anti rat-CD45 Ab, BD Pharmingen). After thawing, cells were used up to four passages, and were then cultured in α-MEM phenol-red free medium, enriched with 20% fetal bovine serum (FBS), 1% penicillin/streptomycin, 1% L-glutamine, and 1% sodium pyruvate, and then incubated at 37 °C and at 5% of CO_2_.

For each sample, a drop of 70 *μ*l containing 45 000 cells was deposited on the substrate, and samples were incubated for 1 h. α-MEM medium was then added to each well to guarantee cell survival for a few days. Analyses were performed on fixed cells 24 h after seeding.

For the single molecule tracking assay, 8000 cells were seeded in an 8-well Lab-Tek Nunc chamber slide in 400 *μ*l of fresh culture medium and maintained in an incubator overnight. A few hours before cell transfection, the cell medium was replaced with 400 *μ*l of antibiotic-free medium (α-MEM, 20% FBS, 1% L-Glutamine).

To investigate reduced internal tension on the Flat condition, before the experiment, MSCs were incubated with 1 *μ*M of cytochalasin-D (Sigma Aldrich, Italy). After 45 min, the solution was replaced with fresh medium and cells were processed for the immunofluorescence assay.

Finally, to evaluate the efficacy of the Nichoid in reducing the transcription factor nuclear import, a transport inhibitor was used. As indicated in Ashkenazy-Titelman (2022), 10 mM puromycin (Thermo-Scientific, Italy) was incubated for 45 min before immunofluorescence experiments.

### D. RNA extraction, library preparation for RNA-Seq, and bioinformatic data analysis

After one week of cell culturing, samples were washed in cold PBS, and the RNA was extracted following the TRIzol Reagent protocol (Invitrogen, USA). RNA quality was assessed using a spectrophotometer (NANOPhotometer^®^ NP80, IMPLEN, USA) and a 2100 Bioanalyzer (Agilent RNA 6000 Nano Kit, Germany). RNAs with a 260:280 ratio of ≥1.5 and an RNA integrity number ≥8 were considered. For RNA-Seq, three samples per condition were analyzed (N = 3). RNA-seq libraries were prepared with the CORALL Total RNA-Seq Library Prep Kit (Lexogen, Austria) using 500 ng total RNA. To remove ribosomal RNA, a RiboCop rRNA Depletion Kit (Lexogen, Austria) was used. The quality of the sequencing libraries was assessed with D1000 ScreenTape Assay using the 4200 TapeStation System (Agilent, USA) and quantified with QubitTM dsDNA HS Assay Kit (Invitrogen, USA). RNA processing was carried out using an Illumina NextSeq 500 Sequencing (Illumina, USA). FastQ files were generated via Illumina bcl2fastq2 (v. 2.17.1.14). A BlueBee^®^ Genomics Platform (Lexogen, Austria) pipeline was used to obtain transcript abundance.

The quality of individual sequences was evaluated using FastQC v. 0.11.9. Adapters were trimmed with Cutadapt, whereas UMIs were removed with UMI tools. Reads were mapped using STAR v. 2.7 with Rattus Norvegicus (Rnor6) as a reference. Transcript intensities were computed using Mix^2^ RNA-Seq Data Analysis Software (Lexogen, Austria) with the “-strandness forward” option. Differential expression analysis was performed using R package DESeq2.

Genes were considered differentially expressed with |log2(Nichoid sample/Flat sample)| ≥ 1 and an FDR ≤ 0.05. R software was used to generate heatmaps (heatmap.2 function from the R ggplots package), dotplots (R ggplots package), and GO Chord plot (GOChord function from the R GOplot package) (Walter *et al.* 2015). Functional enrichment analysis was performed for the Nichoid vs the Flat by considering Kyoto Encyclopedia of Genes and Genomes (KEGG) (https://www.genome.jp/kegg/) and Gene Ontology for cellular component using over-representation analysis (ORA) and gene set enrichment analysis (GSEA). The ORA was performed on g:Profiler web tool (https://biit.cs.ut.ee/gprofiler/gost; Raudvere *et al.*, 2019) using, as input gene list, the list of differentially expressed genes related to mechanotransduction and ranked for decreasing |log2FC|.

All known genes were used as statistical domain scope, and pathways were considered statistically significant if the p-value according to the Benjamin–Hochberg correction was lower than 0.05. GSEA analysis was also performed on iDEP.96 web tool by considering pre-ranked fgsea as the selected method and GO cellular component as selected genesets (Ge *et al.* 2018).

### E. Western blot analysis

Cells were lysed in RIPA lysis buffer (sodium chloride (150 mM), EDTA (5 mM), Tris-HCl (50 mM, pH 8.0), NP-40 (1%), sodium deoxycholate (0.5%), SDS (0.1%), phosphatase, and protease inhibitor cocktail (1%) (Sigma-Aldrich, Italy) for 10 min on ice, then centrifuged at 14 000 rpm for 5 min at 4 °C and supernatants containing the protein soluble fraction were recovered. Samples were heated at 95 °C for 5 min. A total of 15 *μ*g of total soluble protein extract was analyzed by 8% polyacrylamide gel. A 40% Acrylamide/Bis solution 29:1 (Bio-Rad, Italy) was used to handcast 8% polyacrylamide gel. PrecisionPlus^TM^ Protein Dual Color Standards (Bio-Rad, Italy) was used. The gel was then transferred onto a 0.2 *μ*m nitrocellulose membrane (AmershamTM, Germany).

The following antibodies were used: rabbit anti-GAPDH (1:1000, Cell Signaling Technology, Italy), rabbit anti-phospho-lamin A/C (1:1000, Cell Signaling Technology, Italy), mouse anti-lamin A/C (1:2000, Cell Signaling Technology, Italy), and rabbit anti-lamin B1 (1:1000, Novus Biologicals, Italy). Secondary antibodies were goat anti-rabbit and goat anti-mouse conjugated to HRP (Jackson ImmunoResearch, United Kingdom). Chemiluminescent reaction was performed using Clarity^TM^ Western ECL Substrate (Bio-Rad, Italy). Images were acquired using an Alliance imaging system equipped with Nine-Alliance software (UVItec Ltd., Cambridge, UK) and quantified using ImageJ 1.53m (NIH, USA).

### F. Cell transfection and labeling

Two different plasmids were used. The first, pFC27K HaloTag^®^ CMV-neo Flexi^®^ Vector (Promega, ITA), enables cells to produce a small inert molecule called HaloTag^®^ (Promega, ITA). This is an engineered 33 kDa monomeric and catalytically inactive derivative hydrolase protein (England *et al.*, 2015). The second one induces cells to produce the HaloTag^®^ protein to whose N-terminal, the transcription factors MyoD is fused. The MyoD sequence (GenBank reference: M84918.1) was amplified using the forward primer 5′-AGGAGCTAGCATGGAGCTTCTATC-3′ and the reverse primer 5′-TCTCCTCGAGTGAAAGCACCTGATAAATCGC-3′. The Myod sequence was then subcloned in the pFC27K HaloTag^®^ CMV-neo Flexi^®^ Vector using NheI and XhoI cloning sites. The resulting protein is an engineered 70 kDa transcription factor, which covalently binds to permeable vital fluorescent dyes on the HaloTag C-terminal side. This enables live-cell fluorescence imaging to be performed, such as single molecule tracking.

As a plasmid carrier, we used the jet PRIME reagent (Polyplus, USA). A solution consisting of 0.5 *μ*g of DNA, 25 *μ*l of jet PRIME buffer, and 1.12 *μ*l of jet PRIME reagent was prepared and kept at RT for 10 min. The transfection solution was dropped in the cell culture and incubated for 4 h, when it was then replaced with fresh complete medium.

Since the proteins produced after this transfection are not fused with a fluorescent protein, it is necessary to label them in a second step, using the HaloTag^®^-fluorophore. The labeling procedure is summarized as follows: a solution of 0.5 *μ*Μ tetramethylrhodamine (TMR, λ_ex_ = 553, λ_em_ = 575 nm; Thermo Fischer Scientific, Italy) dye was 1:1000 diluted in α-MEM complete medium; 250 *μ*l of labeling solution was placed in each Lab-Teck Nunc well, and the samples were incubated for 30 min. The cells were then washed with 250 *μ*l of phosphate buffered saline (PBS) to remove the unbound fluorescent ligand and incubated with fresh medium for another 30 min, three times. Finally, 1 *μ*g/ml of Hoechst 33342 (Thermo Fischer Scientific, Italy) was used to stain the cell nuclei to obtain a reference image of the cell nucleus before starting the single molecule tracking acquisition of the two proteins HaloTag^®^ and MyoD- HaloTag^®^.

### G. Single molecule tracking imaging acquisition

Experiments were performed on a custom-made microscope with highly inclined illumination, based on an Olympus IX-81 microscope frame, equipped with a 100×, 1.49 NA oil-immersion objective (Olympus Life Science, Segrate, IT) and an EM-CCD camera (Photometrics Evolve 512, Photometrics, Tucson, AZ, USA), for a resulting pixel size of 145 nm. Samples were kept at 37 °C and 5% CO_2_ using an environmental chamber (Okolab SRL, Naples, IT), mounted on a piezoelectric stage for movement in the X and Y directions. A laser source was used for TMR dye excitation (iFlex Mustang, λ = 561 nm, QiOptiq Photonics GmbH, Munich, DE). Movies were collected acquiring 1000 frames with a frame rate equal to 10 ms. As a reference, before each acquisition, an image of the cell nucleus stained with Hoechst 3342 dye was acquired with widefield illumination.

### H. Single molecule tracking imaging analysis

To calculate the radius of curvature (*RC*) along the equatorial nuclear plane of the spread cell, the nuclear envelope was identified using the reference image stained with Hoechst 3342. Drawing a manual ROI, the nuclear envelope was identified and set with a 1-pixel thickness, and its coordinates were obtained. For a more precise calculation, an interpolation with a cubic spline was performed, and the RC values were then calculated along the spline. The curvature along the equatorial nuclear plane *κ*, i.e., the inverse of the radius of curvature (*κ = RC* **^−^**^1^), of a parametric curve $\gamma \left(s\right)=[x\left(s\right),\;y\left(s\right)],$ can be calculated as

$$\kappa =\frac{\left|\dot{x}\ddot{y}-\dot{y}\ddot{x}\right|}{{\left({\dot{x}}^{2}+{\dot{y}}^{2}\right)}^{3/2}},$$ (1)

where x and y are real-valued differentiable functions, and $\dot{x},\dot{y,}$ and $\ddot{x}$, $\ddot{y}$ are the first and second derivatives of the parametric curve with respect to the arc length *s*.

Single molecule movies were analyzed with the TrackMate-ImageJ plug-in (Tinevez *et al.,* 2017). A Gaussian filter was applied to smooth images and, subsequently, a Laplacian filter was used to highlight regions with rapid fluorescence intensity changes. The detection was established by setting the estimated particle diameter as 1 *μ*m, the acquisition time *t* = 10 ms, and the maximum distance reachable over a time step $r_{\text{max}}=1\;\mu m$. At the end of the analysis, the program returns tracks (a list of X–Y coordinates) of each analyzed molecule. Of all the tracks, only those in a 5-pixel neighborhood close to the defined nuclear edge were considered. In this region, 30 000 and 23 000 single-molecule jumps were identified and analyzed for HaloTag^®^-MyoD and HaloTag^®^, respectively.

Finally, the mobility of individual molecules translocating from the cytosol to the nucleus through the nuclear envelope was investigated as a function of the nuclear radius of curvature (*RC*). Using a custom-made MATLAB code, the percentage of detected events was calculated (fraction of molecules translocating into the nucleus) as a function of the nuclear envelope RC. The results are summarized in Fig. 2(c).

### I. Immunofluorescence assay

After removing the medium, the samples were rinsed at least twice with PBS and fixed for 15 min in paraformaldehyde solution (PAF, 4%). To remove PAF residues and to diminish cell autofluorescence, the samples were washed three times with glycine 0.1 M solubilized in PBS. Cells were permeabilized with 0.25% Triton-X-100 in PBS solution for 10 min. In order to block nonspecific binding of the antibodies, the cells were then incubated at room temperature with a solution of 2% bovine serum albumin (BSA) 2% and 0.1% Tween in PBS for a few hours (up to 4).

Depending on the proteins to be observed, samples were incubated overnight at 4 °C with primary antibodies diluted in 2% BSA + 0.1% Tween in PBS [Anti-Histone H3 trimethyl K4 (Abcam, UK), Anti-Histone H3 trimethyl K9, Anti-lamin A/C, Anti-phospho-lamin A/C, (Lamin A/C phosphorylated at serine 22, Cell Signaling Technology, USA), C-myc (Santa Cruz, USA), PPAR-g (Cell Signalling, USA)]. The next day, samples were rinsed three times with 0.1% Tween in PBS and then incubated at room temperature, in the dark, for 45 min with a solution of 2% BSA + 0.1% Tween in PBS containing secondary antibodies (Anti-Rabbit IgG H&L/Anti-Mouse IgG H&L conjugated with Alexa Fluor^®^488 or Alexa Fluor^®^647, Abcam, Italy) and, where necessary, Phalloidin-FITC dye (Sigma Aldrich-Merck, Italy). After washing three times with 0.1% Tween in PBS, cell nuclei were labeled with Hoechst 33342. Finally, samples were rinsed twice more with PBS and water, to remove salts contained in PBS, before mounting them with a drop of Fluor Save mounting medium (Sigma-Aldrich, Italy) on microscope slides.

#### 1. Confocal microscopy image acquisition

The nuclear morphology, chromatin organization, and actin cytoskeleton were analyzed on fluorescence images acquired by a confocal microscope Nikon Ar1^+^ equipped with four wavelength diode lasers (λ_excitation_ = 405/488/561/640 nm).

Stained cells were imaged with a 60× oil immersion objective, with 1.4 NA 0.13WD. The pinhole was set to 0.9 Airy Unit. 1024 × 1024 pixel images were acquired as *z-*stack images. Cells grown on the Flat as well as in the Nichoid were imaged with a 1 *μ*m step, resulting in an acquisition depth of approximately 10 *μ*m for the former, and a depth of approximatively 40 *μ*m for the latter.

#### 2. Nuclear morphology and actin organization

After staining the cell nucleus with Hoechst 33342, the size of the nucleus was determined using the *z-*stack images acquired by confocal microscopy with a 0.5 *μ*m step. The cell nucleus was modeled by ellipsoids, in which semiaxes *a–c* were measured using the orthogonal projections of the *z-*stack image acquisitions (Jacchetti *et al.*, 2021). These semiaxes values were used to create ellipsoids (using GNUplot software) that were representative of the nuclear shape of the two investigated cell populations. The Gaussian curvature radius along the equatorial nuclear plane was calculated on these ellipsoids (Bektas, 2017)—see Figs. 1(d) and 2.

To measure the nuclear curvature at the level of the cellular equatorial plane, we manually drew a ROI around each nuclear edge, and its coordinates were obtained. An interpolation with a cubic spline was performed, and the radius of curvature was calculated, as described earlier “Single molecule tracking imaging analysis.” A frequency distribution analysis of the nuclear radius of curvature (*RC*) is reported in Fig. 3(a).

Actin cytoskeleton directionality analysis around the cell nucleus was performed creating an *xy*-projection of each *z-*stack image and manually drawing a rectangular ROI circumscribing each cell nucleus. The Fiji (https://imagej.net/software/fiji/.net) “Directionality” was used to compute a directionality histogram indicating the amount of actin fibers in each direction (from −90° to +90°, step 1°). Images of structures with a preferred orientation returned a sharp peaked histogram, while images of isotropic structures produced a smoothed histogram. To create a Gaussian representative fitting of actin fiber directionality, for each cell, we shifted the highest peak of the histograms at 0° x-axis. The fit was calculated using OriginPro (https://www.originlab.com/2021).

#### 3. Internalization of transcription factors

After the immunofluorescent assay in which cells were stained with Hoechst33342 dye and the anti PPAR-g and C-Myc antibodies, respectively, to visualize the cell nucleus and the cytoplasmic zone, nuclear vs cytosol intensity was analyzed. The Hoechst33342 was used to identify the nucleus and to draw a ROI close to the edge. The cytoplasmic ROI was drawn outside the nucleus with a dimension as similar to possible to the previous one. The transcription factor internalization was analyzed by calculating the ratio between the fluorescence intensity inside the nucleus and in the cytoplasm.

#### 4. Chromatin homogeneity analysis

Chromatin organization was evaluated using fluorescence images where all the DNA (Hoechst 33342 dye) and euchromatin (H3K4me3) were stained.

To investigate the spatial distribution homogeneity of the euchromatin in the equatorial plane of cells, we drew a linear ROI from the nuclear edge toward the nuclear center. To prevent artifacts in the measurements, the DNA-free nucleoli were avoided. For each cell, we obtained the intensity profile as gray values of the pixel along the selected ROI. From each intensity profile, the mean intensity ( $\bar{I}$) and its standard deviation ( $\sigma_{I}$) were calculated in order to evaluate the homogeneity of the distribution using the dimensionless parameter CV,

$$CV=\frac{\sigma_{I}}{\bar{I}}\;[\;].$$ (2)

#### 5. Lamin A/C and phospho-lamin A/C distribution analysis

The images acquired enabled us to investigate the distribution and organization of lamin A/C and phospho-lamin A/C (Stiekema *et al.*, 2021). Three planes in the *z-*coordinate were analyzed for each cell nucleus: the bottom plane, equatorial plane, and top plane [see Fig. 5(e), right].

For each plane, two ROIs were drawn: a segmented line to outline the border of the nucleus, and a polygon selection that comprised the internal part of the nucleus within a 0.5 *μ*m distance (equal to 5 pixels) from the border. The mean intensity $\bar{I}$ and its standard deviation $\sigma_{I}$ were measured for each ROI. With these data, the dimensionless parameter “β,” defined as the ratio between the mean intensity along the edge and the mean intensity inside the nucleus, was calculated as

$$\beta =\frac{{\bar{I}}_{\text{edge}}}{{\bar{I}}_{\text{inside}}}\;[\;],$$ (3)

where β represents the quantity of proteins found at the nuclear edge compared to those found in the nucleoplasm.

To investigate the uniformity and organization of lamin A/C and phospho-lamin A/C along the nuclear edge at the equatorial plane of the cell nucleus, we calculated the fluorescence intensity coefficient of variation *C*V [see Eq. (2)], where $\bar{I}$ is the mean intensity calculated on a ROI corresponding to the nuclear edge, and $\sigma_{I}$ corresponds to the relative standard deviation.

To investigate the lamina thickness and distribution of lamin A/C in the nuclear interior, for each cell, a linear ROI corresponding to the nuclear major axis was drawn at the equatorial plane of the nucleus. Then, in order to compare all curves, the cell diameters were normalized to 1, and the intensity profile $I_{i}$ of each ROI was measured and normalized to the maximum intensity value *I*_max_ of the related dataset,

$${\tilde{I}}_{i}=\frac{I_{i}}{I_{\text{max}}}\;[\;].$$ (4)

The resulting curve ${\tilde{I}}_{i}$ is a downward sloping curve composed of two functions, the first representing the intensity decrease close to the nuclear edge, and the second representing the distribution of the fluorescence intensity inside the nucleus. We were mainly interested in the part close to the nuclear edge; therefore, this portion of the curves was fitted linearly to calculate the corresponding slope [Fig. 5(a)]. Lamina thickness was estimated as the mean and standard deviation from the average of each calculated slope (n = 47, 47, and 39 for Flat, Flat+CD, and Nichoid, respectively).

To quantify the correlation between lamin A/C and phospho-lamin A/C in the nucleus and to visualize the protein distribution in space, floating point images of the correlation map were acquired with NIS-Elements (Nikon). Data were thresholded with a custom-made MATLAB code to eliminate correlation values ranging from −0.4 to +0.4 [Fig. 5(f)]. We used the other values to calculate the percentage of correlation inside the nucleus and at the nuclear edge, and to describe the relationship between the nuclear curvature and the number of correlating pixels in proximity to the nuclear envelope [Fig. 5(g)].

### J. Lamin A/C and phospho-lamin A/C computational analysis

The structural model of the CC1b lamin domain (79–222 residues) was obtained from the structure of the longer lamin domain available in the Protein Data Bank (code 6JLB) [Fig. 6(b)]. For protein visualization, the VMD server was used. The CC1b tetramer domain was solvated in a water box, thereby ensuring a 5 Å layer of water in each direction. The final solvated all-atom system contained 169 084 atoms, including 159 660 water atoms.

The first step of energy minimization was carried out using NAMD software and the CHARMM force field. Full atomistic simulation was performed by ACEMD3, in explicit solvent conditions. To constrain the covalent bond lengths, rigid bonds were used with an integration time step of 4 fs. Nonbonding interactions were computed using a cutoff for the neighbor list at 9 Å, with a switching distance of 7.5 Å for van der Waals interactions. The particle-mesh Ewald summation (PME) method was used to describe electrostatic interactions. Periodic boundary conditions were imposed. The tetrameric lamin model was equilibrated through 570 ns NVT molecular dynamic simulations at a temperature of 300 K and with 1 bar pressure.

Structural convergence was evaluated based on root mean square deviation (RMSD) analysis via a VMD server. To investigate the differences between the monomer, dimer, and tetramer, H-bonds were extracted by the VMD server at each frame of the last 70 ns of stable RMSD regime, with a donor–acceptor distance smaller than 0.3 nm, and donor–hydrogen-acceptor angle smaller than 20°. For each H-bond, the percentage occurrence was calculated, and only H-bonds with a percentage occurrence higher than 20% were considered.

In the stable RMSD regime, the CC1b lamin model was extracted from three different frames randomly selected in the last 3 ns of the equilibration trajectory. For each frame, three different molecular arrangements were extracted: the tetramer (T arrangement, which consists of chains A, B, C, and D), the dimer (D arrangement, which consists of chains A and B), and the monomer (chain B). Constant-force-steered molecular dynamic (SMD) simulations were performed to reproduce creep test conditions where a constant force is applied, and the molecule rearranges itself to the load applied. Generalized Born implicit solvent and the CHARMM force field were used in NAMD software. The temperature was set to 310 K, and the time step to 2 fs.

Tensile deformation was simulated by fixing N-terminal Cα atoms (residue 79) of each chain of the specific arrangement and applying an instantaneous step force of 140 pN (2 Kcal/mol/Å) to the C-terminal Cα atoms (res 222) of each chain. To define the force direction, all the configurations were reduced to a single “equivalent chain” representative of the average orientation of the whole structure. Equivalent chain N-terminal and C-terminal coordinates were calculated by averaging the Cα atom coordinates of residues 79 and 222, respectively. The pulling force direction was therefore calculated in accordance with the equivalent chain direction. Force was applied as independent vectors to each chain of the M, D, and T arrangements. The force was maintained until the asymptotic deformation was reached.

For each chain, we estimated the equivalent chain viscoelastic characteristics as being representative of the whole lamin model. The equivalent chain steered atom displacement vs time curve was used to extract mechanical parameters. The engineering strain ε was obtained from the measured displacement (ΔL/L_0_) over time, where L_0_ is the chain length for σ = 0. The Kelvin–Voigt model was used to fit computational data to estimate Young's modulus E and the viscosity η parameters by MATLAB code from the following equations:

$$\varepsilon \left(t\right)=\frac{\sigma_{0}}{E}(1-e^{-\frac{E\cdot t}{\eta }}),$$ (5)

$$\sigma_{0}=\frac{F}{A},$$ (6)

where σ_0_ represents the instantaneous stress, which was calculated by normalizing the force F by the cross-sectional area A. In line with the decision to reduce all the configurations to the single equivalent chain, the cross-sectional area refers to the single α-helix structure characterized by an internal diameter of 5 Å (A = 78.54 Å^2^). For each arrangement, the three different frames randomly selected in the last 3 ns of the equilibration trajectory were averaged to obtain a single strain value representative of the specific test conditions.

### K. Statistical analysis

In order perform a solid statistical analysis, all the experiments were independently repeated at least three times per substrate: Flat, the Nichoid, and both the samples treated with 1 *μ*M cytochalasin-D. The total number of cells analyzed in the immunofluorescence assay varied according to the element investigated. The analysis of actin cytoskeleton was performed on 214, 140, and 73 cells, respectively (Flat, Flat+CD, Nichoid). For nuclear morphology and chromatin spatial distribution, there were 235, 193, and 120 cells analyzed. For the evaluation of the nuclear level of transcription factors, 212, 188, 96, 164, 113, and 128 cells were analyzed for the following conditions: Flat, Flat+CD, Flat+Puro, Nichoid, Nichoid+CD, Nichoid+Puro. In lamin/phospho-lamin A/C characterization, 56, 57, 55, and 49 cells were analyzed, respectively.

Finally, in single molecule tracking investigations, around 20 cells spread on the glass coverslip, including 30 000 and 23 000 single-molecule jumps, were identified and analyzed in the two populations HaloTag^®^-MyoD and HaloTag^®^, respectively. Statistical analyses were performed with OriginPro. In assays comparing just two cell populations (the Flat and the Nichoid), we used the non-parametric Mann–Whitney statistical test. In the analyses comparing three different samples (Flat, Flat+CD, and Nichoid), the non-parametric Kruskal–Wallis test was used.

## SUPPLEMENTARY MATERIAL

See the supplementary material for complementary figures and tables as support and insight of data presented in the main manuscript.

## Data Availability

The data that support the findings of this study are available from the corresponding authors upon reasonable request.
